# Single extracellular vesicle surface protein‐based blood assay identifies potential biomarkers for detection and screening of five cancers

**DOI:** 10.1002/1878-0261.13586

**Published:** 2024-01-09

**Authors:** Yuxin Min, Wenjiang Deng, Huangbo Yuan, Dongliang Zhu, Renjia Zhao, Pengyan Zhang, Jiangli Xue, Ziyu Yuan, Tiejun Zhang, Yanfeng Jiang, Kelin Xu, Di Wu, Yanling Cai, Chen Suo, Xingdong Chen

**Affiliations:** ^1^ Department of Epidemiology, School of Public Health Fudan University Shanghai China; ^2^ Department of Medical Epidemiology and Biostatistics Karolinska Institute Stockholm Sweden; ^3^ State Key Laboratory of Genetic Engineering, School of Life Science Human Phenome Institute, Fudan University Shanghai China; ^4^ Fudan University Taizhou Institute of Health Sciences Taizhou China; ^5^ Yiwu Research Institute of Fudan University China; ^6^ Department of Biostatistics, School of Public Health Fudan University Shanghai China; ^7^ Vesicode AB Stockholm Sweden; ^8^ Guangdong Provincial Key Laboratory of Systems Biology and Synthetic Biology for Urogenital Tumors, Shenzhen Key Laboratory of Genitourinary Tumor, Department of Urology The First Affiliated Hospital of Shenzhen University, Shenzhen Second People's Hospital, Shenzhen Institute of Translational Medicine Shenzhen China; ^9^ Shanghai Institute of Infectious Disease and Biosecurity Shanghai China; ^10^ State Key Laboratory of Genetic Engineering, Human Phenome Institute, Zhangjiang Fudan International Innovation Center, and National Clinical Research Center for Aging and Medicine, Huashan Hospital Fudan University Shanghai China

**Keywords:** biomarkers, EV subpopulations, extracellular vesicle proteins, pan‐cancer

## Abstract

Extracellular vesicles (EVs) and EV proteins are promising biomarkers for cancer liquid biopsy. Herein, we designed a case–control study involving 100 controls and 100 patients with esophageal, stomach, colorectal, liver, or lung cancer to identify common and type‐specific biomarkers of plasma‐derived EV surface proteins for the five cancers. EV surface proteins were profiled using a sequencing‐based proximity barcoding assay. In this study, five differentially expressed proteins (DEPs) and eight differentially expressed protein combinations (DEPCs) showed promising performance (area under curve, AUC > 0.900) in pan‐cancer identification [e.g., TENM2 (AUC = 0.982), CD36 (AUC = 0.974), and CD36‐ITGA1 (AUC = 0.971)]. Our classification model could properly discriminate between cancer patients and controls using DEPs (AUC = 0.981) or DEPCs (AUC = 0.965). When distinguishing one cancer from the other four, the accuracy of the classification model using DEPCs (85–92%) was higher than that using DEPs (78–84%). We validated the performance in an additional 14 cancer patients and 14 controls, and achieved an AUC value of 0.786 for DEPs and 0.622 for DEPCs, highlighting the necessity to recruit a larger cohort for further validation. When clustering EVs into subpopulations, we detected cluster‐specific proteins highly expressed in immune‐related tissues. In the context of colorectal cancer, we identified heterogeneous EV clusters enriched in cancer patients, correlating with tumor initiation and progression. These findings provide epidemiological and molecular evidence for the clinical application of EV proteins in cancer prediction, while also illuminating their functional roles in cancer physiopathology.

## Graphical abstract

The study provided the molecular characterization of plasma‐derived extracellular vesicle (EV) surface proteins between patients with one of five cancers and controls. In bulk analysis, we found several potential EV protein biomarkers for pan‐cancer. Furthermore, using the protein expression data at the single EV level, we identified EV subpopulations enriched in cancer or control samples, unveiling the heterogeneity of EVs.

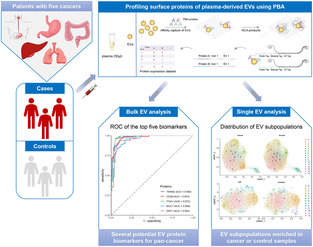

**Abbreviations**
ADAM10: A disintegrin and metalloproteinase 10
AFP: alpha‐fetoprotein
ANXA1: annexin A1
AUC: area under curve
CA19‐9: carbohydrate antigen 19–9
CADM3: cell adhesion molecule 3
CAV1: caveolin 1
CDH13: cadherin 13
CEA: carcinoembryonic antigen
cfDNA: circulating free DNA
CR: colorectal cancer
CTB: cholera toxin subunit B
CTCs: circulating tumor cells
ctDNA: circulating tumor DNA
DEPCs: differentially expressed protein combinations
DEPs: differentially expressed proteins
DSC1: desmocollin 1
EGFR: epidermal growth factor receptor
EPCAM: epithelial cell adhesion molecule
ES: esophageal cancer
ESAM: endothelial cell adhesion molecule
EVs: extracellular vesicles
FN1: fibronectin 1
FPR: false‐positive rate
GO: Gene Ontology
GPA33: glycoprotein A33
GPI: glycosyl‐phosphatidyl‐inositol
HAVCR2: hepatitis A virus cellular receptor 2
ILK: integrin‐linked kinase
ITGA*: integrin subunit alpha*
ITGB*: integrin beta*
KEGG: Kyoto Encyclopedia of Genes and Genomes
KIT: KIT proto‐oncogene; receptor tyrosine kinase
L1CAM: L1 cell adhesion molecule
LAG3: lymphocyte activating 3
LAMP1: lysosomal‐associated membrane protein 1
LGR5: G‐protein‐coupled receptor
LI: liver cancer
LU: lung cancer
MISEV2018: minimal information for studies of extracellular vesicles 2018
MUC1: mucin 1
MUC4: mucin 4
NES: nestin
NLGN1: neuroligin 1
NPV: negative predictive value
nt: nucleotide
NTA: nanoparticle tracking analysis
PBA: proximity‐dependent barcoding assay
PCA: principal component analysis
PPI: protein–protein interaction
PPV: positive predictive value
PTPRJ: protein tyrosine phosphatase receptor type J
RCA: rolling circle amplification
ROC: receiver‐operating characteristic
SEM: scanning electron microscope
SIGLEC10: sialic acid‐binding Ig‐like lectin 10
ST: stomach cancer
TENM2: teneurin transmembrane protein 2
TIMP2: TIMP metallopeptidase inhibitor 2
TPR: true‐positive rate
UMI: unique molecular identifier
VCAM1: vascular cell adhesion molecule 1

## 1 Introduction

Cancer is a major global public health issue. e of almost all cancers, survival rate can significantly increase on early detection and prompt treatment [1]. As reported by the Office for National Statistics UK, the 5‐year survival rate of lung cancer at stage IV is only 2.9%, but at stage I, it is as high as 56.6%; similarly, for colorectal cancer, the survival rate increases to > 80% when it is diagnosed at stage I or II, in comparison to only 10.3% at stage IV [2]. Thus, cancer detection at an earlier point is vital to treatment outcomes. Cancer screening, which involves a set of medical procedures intended to identify the presence of cancer and treat it properly before any symptoms appear or conventional clinical diagnosis, is an important strategy to alleviate disease burden [1, 3]. However, there are various limitations in the existing cancer screening tools such as endoscopy being an invasive procedure, unsuitable for population‐scale screening or continuous monitoring, and traditional tumor markers, including alpha‐fetoprotein (AFP), carcinoembryonic antigen (CEA), and carbohydrate antigen 19‐9 (CA19‐9), lacking sensitivity and specificity at early stages [4]. Liquid biopsy, involving cancer diagnosis by assessing human biofluids (e.g., blood, saliva, and urine), has drawn increasing attention in the realm of cancer screening and progression monitoring [5]. Relative to common approaches, liquid biopsy has the advantage of minimum invasiveness, continuous accessibility, and better sensitivity and specificity, applicable to large‐scale population‐based cancer screening projects [6]. In general, circulating tumor cells (CTCs), circulating tumor DNA (ctDNA), and extracellular vesicles (EVs) represent the most popular biomarkers in liquid biopsy [7].

Extracellular vesicles are bi‐layered nanovesicles secreted by diverse living cells, with no replication capability [8]. They carry nucleic acids, lipids, and proteins from donor cells, mediating intercellular transportation and communication under both physiological and pathological conditions [9, 10, 11]. EV proteins reportedly play a key role in tumorigenesis and disease progression by suppressing immune response, promoting angiogenesis, and preconditioning sites for metastasis [9, 12, 13]. In comparison with CTCs and ctDNA, EVs possess the edge of high stability, wide distribution, and high quantities in biofluids [7, 14]; accordingly, they represent ideal biomarkers for cancer signal detection in clinical applications. For instance, Melo et al. [15] conducted a cohort study and reported that glypican 1 expression level in serum EVs could distinguish between patients with pancreatic cancer and controls (area under curve, AUC = 1.000). Subsequently, the potential of glypican 1 in EVs as a cancer diagnostic biomarker was also confirmed by other research groups in different populations [16, 17, 18], contributing to the gradual emergence of rapid diagnostic methods for pancreatic cancer. Park *et al*. found that the combination of epidermal growth factor receptor (EGFR), epithelial cell adhesion molecule (EPCAM), CD24, and glycoprotein A33 (GPA33) in EVs could discriminate patients with colorectal cancer from controls (AUC = 0.980) [19]. Several other studies have also reported the potential of EV proteins to detect other cancer types [9, 14]. However, most previous studies only focus on one cancer type, implying that the identified biomarker is not tested for cancer type specificity via comparison among different cancers. Furthermore, features shared across diverse cancers have been studied from multiple aspects, such as genetic mutations, pathogenic pathways, and immune signatures [20]; however, the EV biomarkers for pan‐cancer diagnosis have not received widespread recognition. Hinestrosa et al. [21] used an EV protein‐based blood test for multicancer detection, which showed good effectiveness in distinguishing controls from patients with pancreatic, ovarian, or bladder cancer. Nevertheless, studies based on EV proteins and high‐incidence cancers remain scarce [22].

With the aim of detecting potential common and type‐specific cancer biomarkers, we profiled surface proteins of circulating EVs in plasma using a proximity‐dependent barcoding assay (PBA) [23] to analyze differences in protein expression levels between controls and patients with high‐incidence cancers, namely esophageal, stomach, colorectal, liver, and lung cancer; moreover, their classification performance was evaluated. Besides, we utilized bioinformatic tools to elucidate the biological functions of EV proteins in tumorigenesis and disease progression. A list of EV surface proteins with promising performance in differentiating patients with high‐incidence cancers from controls is reported.

## 2 Materials and methods

### 2.1 Study design and population

**Fig. 1:**
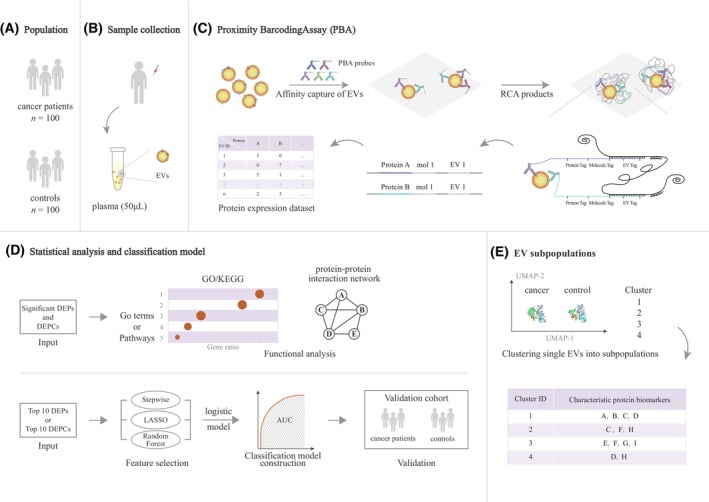
Schematic diagram of the study design and PBA workflow. (A) The discovery dataset (*n* = 200) comprised 100 cancer patients and 100 controls from Taizhou. (B) For each sample, 50 μL plasma was collected to profile EV surface proteins. (C) PBA probes were generated by chemical conjugation of antibodies and DNA oligonucleotides, which included 8‐nt protein tag to specify each protein and 8‐nt UMI tag to denote each unique molecule. RCA products containing 15‐nt EV tag were produced to specify single EV. DNA oligonucleotides on PBA probes were then hybridized to a unique RCA product, followed by enzymatic extension, to incorporate the EV tag to the protein and UMI tag. The combined motif was then sequenced and sequencing reads were analyzed to quantify EV protein expression. (D) Statistical analysis was applied to identify significant DEPs and DEPCs between cancer and control samples. GO, KEGG, and protein–protein interaction analyses were performed to reveal potential molecular functions. Stepwise bidirectional elimination, Lasso, and Random Forest algorithm were implemented to select optimal biomarkers and a logistic model was constructed to evaluate the classification power of EV proteins. The performance was then validated in a validation dataset. (E) EV subpopulations and cluster‐specific proteins were detected using the Seurat clustering package. [Correction added on 16 February 2024 after first online publication: Figure 1C was replaced due to an error in the schematic representation of the rolling circle amplification (RCA) products.]

We designed a hospital‐based case–control study involving 100 patients (71 men and 29 women, median age = 65 years) diagnosed with esophageal (*n* = 20), stomach (*n* = 20), colorectal (*n* = 20), liver (*n* = 20), or lung (*n* = 20) cancer at Taizhou People's Hospital from August 2008 to November 2012. They were randomly sampled from patients diagnosed with the five cancers for the first time during this period. Blood samples were collected from all patients by professional health workers. At the time of sample collection, 100 sex‐ and age‐matched controls (71 men and 29 women, median age = 65 years) without any history of cancer were selected from the Taizhou longitudinal cohort, which has been comprehensively described in a previous study [24]. All enrolled subjects were Han residents in Taizhou, Jiangsu province, China. Given the sample size, it was sufficient to verify that a potential biomarker had an expected AUC value of 0.800 to distinguish between patients with cancer and controls, with a power of 90% and a significance level of 0.05. PASS v15 was used for sample size calculation [25]. Additionally, we recruited 14 patients with esophageal (*n* = 2), stomach (*n* = 4), colorectal (*n* = 4), liver (*n* = 2), or lung (*n* = 2) cancer and 14 controls as a validation dataset from the same source. For differentiation, the aforementioned 200 subjects were referred to as the discovery dataset (Fig. 1A). Unless otherwise specified, the analyses in this study were conducted based on the discovery dataset. Detailed demographic and epidemiological information is provided in Table S1. This study was approved by the Human Ethics Committee of Fudan University Taizhou Institute of Health Sciences (EC_AF_006). Written informed consent was obtained from all subjects prior to their inclusion in the study. The study methodologies conformed to the standards set by the Declaration of Helsinki.

### 2.2 Plasma sample processing and EV characterization

Blood samples were collected in a K2 EDTA vacutainer and centrifuged (TDZ5‐WS centrifuge). Plasma was separated and aliquoted into a barcoded cryovial for long‐term storage at −80 °C in the biobank of Fudan University Taizhou Institute of Health Sciences. For each subject, 50 μL plasma was used for EV capture and EV surface protein profiling (Fig. 1B). EV morphology, size distribution, and protein markers were characterized according to Minimal Information for Studies of Extracellular Vesicles 2018 (MISEV2018) guidelines [8]. Ultracentrifugation with 100 000 ***g*** for 70 min (Beckman, Brea, CA, USA, XPN100) was performed to purify EVs for nanoparticle tracking analysis (NTA, NanoSight NS300, Malvern Panalytical, Worcestershire, UK). EVs captured in the following PBA assay well were fixed with 4% formaldehyde and imaged with a scanning electron microscope (SEM, Hitachi SU8010, Tokyo, Japan) with the electron beam at a voltage of 60 KV and the secondary electron detector. Detection of EV markers including transmembrane or glycosyl‐phosphatidyl‐inositol (GPI)‐anchored protein (CD9, CD63, CD81, etc.) and cytosolic protein with membrane‐binding or association capacity (annexin A1, ANXA1; Caveolin 1: CAV1, etc.) was included in the multiplexed profiling panel of PBA.

### 2.3 Profiling EV surface proteins with PBA

To profile single EV surface proteins, PBA was performed using antibody–DNA conjugates and next‐generation sequencing as described in our earlier study [23]. PBA probes were prepared by attaching antibodies to DNA oligonucleotides, consisting of an 8‐nucleotide (nt) protein tag, which identifies the specific EV surface protein, and an 8‐nt random unique molecular identifier (UMI) sequence known as molecular tag, responsible for distinguishing individual protein molecules after PCR amplification and DNA sequencing. To barcode single EVs, circularized DNA molecules containing a 15‐nt random DNA sequence, referred to as complex tags, were subjected to rolling circle amplification (RCA). These complex Tags were integrated into the antibody‐conjugated oligonucleotides, serving as markers for proteins on single EVs that have colocalized with unique RCA products.

As Fig. 1B,C showed, EVs were captured in microtiter wells coated with cholera toxin subunit B (CTB), which bound to GM1 gangliosides on EV membranes. After the washing step with PBS, unbound components were removed. PBA probes were added to each well with captured EVs. Subsequent washes eliminated unbound antibody conjugates. Then, we introduced diluted RCA products, which enabled the interaction between individual RCA products (comparable in size to EVs) and single EVs. Through DNA polymerase‐mediated extension, PBA probes bound to the same EV incorporate the same complex Tag from a nearby RCA product. The extension products were amplified for constructing a sequencing library and a sample tag was also added to each sequence to mark different sample origins. The high‐throughput sequencing was performed on the Illumina NextSeq 500 platform. Subsequently, data from sequencing reads, which comprised protein tag, EV tag, UMI, and sample tag, were transferred into sample ID, EV ID, protein type, and protein quantity, respectively. A total of 207 EV surface proteins were profiled using this PBA (Table S2), consisting of EV markers (CD9, CD63, CD81, ANXA1, CAV1, etc.), disease markers, and cellular adhesion molecules.

### 2.4 Quality control of EV surface protein data and batch effect adjustment

Raw sequencing data, produced in BCL format, were converted into fastq files using bcl2fastq (v2.20.0.42, Illumina Inc., San Diego, CA, USA). fastqc [26] was employed to evaluate data quality; reads with quality scores < 20 were removed. These clean data were then processed to obtain a high‐dimensional protein expression dataset at a single EV level. For batch effect adjustment of samples assayed in different 96‐well plates, we applied a negative binomial regression model based on the ComBat‐seq package [27], which can retain the integer nature of EV protein expression data.

### 2.5 Statistical analysis

Statistical analyses were performed using r (v4.0.5) and results with *P*‐value < 0.05 were considered significant. Protein expression values were normalized using the trimmed mean of M‐values algorithm in the edgeR package [28] to eliminate the influence of different library sizes. We applied Student's *t*‐test or Wilcoxon rank‐sum test to compare differences in protein expression between the sample groups, and *P*‐values were adjusted with the Benjamini–Hochberg method (*P*‐adj). To assess the performance of differentially expressed proteins (DEPs) in distinguishing between patients with cancer and controls, receiver‐operating characteristic (ROC) curves for all potential biomarkers were plotted and AUC values were calculated using pROC (v1.18.0) [29].

### 2.6 Functional analysis using bioinformatic tools

Gene ontology (GO), Kyoto Encyclopedia of Genes and Genomes (KEGG), and protein–protein interaction (PPI) analyses were performed to reveal potential molecular functions of all DEPs including the upregulated and downregulated, to identify pertinent biological pathways, and to assess interactions among proteins, respectively. PPI was analyzed by string 11.5 [30], a web‐based tool (http://string‐db.org), and results were visualized using cytoscape 3.9.1 [31].

### 2.7 Analysis of protein combinations and subpopulations at the single EV level

The PBA approach profiled protein expression at a single EV level, generating novel EV‐related data to investigate the heterogeneity of EVs. We assessed the co‐expression of proteins as protein combinations on each EV. ROC analysis was performed to evaluate the classification performance of all differentially expressed protein combinations (DEPCs). Furthermore, single EV data were clustered into different subpopulations using the Seurat package (v4.0.6) [32]. We also analyzed differences between cancer and control samples within each EV cluster using binomial test, and subpopulation‐specific proteins with potential classification significance were identified.

### 2.8 Classification model construction and internal cross‐validation

To further evaluate the classification potential of DEPs and DEPCs, we leveraged logistic regression to distinguish between patients with cancer (*n* = 100) and controls (*n* = 100). We used Stepwise bidirectional elimination, Lasso, and Random Forest algorithms to select optimal biomarkers. The protein biomarkers that remained after stepwise bidirectional and Lasso selection, as well as the top half of the biomarkers ranked by variable importance in Random Forest analysis, will be used to construct the logistic regression model if they are selected by at least two algorithms. Ten‐fold cross‐validation was applied to evaluate the model performance and the mean AUC value of test sets was reported.

We also attempted to distinguish one type of cancer (*n* = 20) from the other four types of cancer (*n* = 80) using DEPs or DEPCs. Similarly, we included the top 10 DEPs between groups in a stepwise bidirectional variable selection process. We then constructed cancer‐type‐specific models based on the selected variables using logistic regression. Considering the small number of samples in each group, we applied leave‐one‐out cross‐validation [33] approach to measure the performance of each classification model. In this approach, one sample was left out as validation data, and the remaining samples were utilized as training data to build the classification model. The process was repeated for all samples and overall accuracy of the model was recorded.

### 2.9 Classification model performance in the validation dataset

To validate the classification performance of the constructed model in distinguishing cancer patients from controls, we used the additional 28 samples as the validation dataset. We calculated the AUC value for the validation dataset and conducted a *post hoc* analysis to evaluate the statistical power in this small‐sample validation using pass v15 [34, 35]. The time interval between the implementation of PBA test on the validation dataset and the discovery dataset was 1 year.

The aforementioned methods were illustrated by the schematic diagram of the study design and PBA workflow (Fig. 1).

## 3 Results

### 3.1 Characterization of EVs and surface proteins detected in all plasma samples

**Fig. 2:**
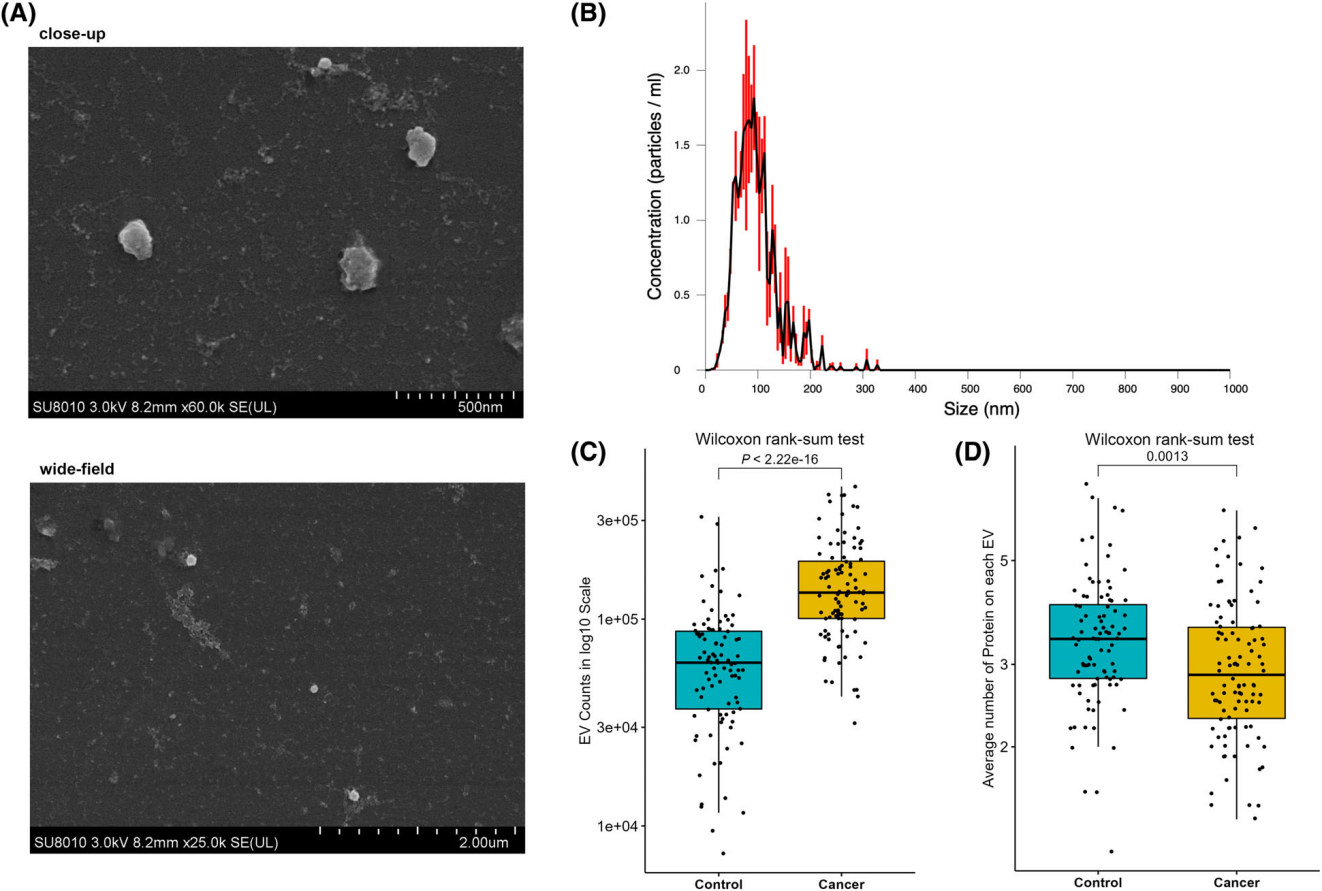
Characterization of EVs detected in plasma. (A) Morphology of EVs captured by scanning electron microscopy, both in close‐up (scale bar indicates 500 nm) and wide‐field (scale bar indicates 2.00 μm) views. (B) Diameter distribution of EVs obtained by nanoparticle tracking analysis method. (C) Comparison of EV counts between pan‐cancer (*n* = 100) and control (*n* = 100) groups, *P* < 0.001. (D) Comparison of the average number of proteins on each EV between pan‐cancer (*n* = 100) and control (*n* = 100) groups out of the 207 possibly detectable proteins by PBA, *P* < 0.01.

Extracellular vesicle properties were characterized using SEM and NTA methods (Fig. 2A,B). The average diameter of captured EVs was 96.90 ± 3.00 nm and the mean concentration was 1.73e + 10 particles·mL^−1^. For each sample, the average number of EV counts and detected proteins were 1.15e + 5 and 3.48e + 5, respectively. The median count of EVs was 134 291 in cancer samples and 61 569 in controls (*P* < 0.001, Fig. 2C). Of 207 possibly detectable proteins by PBA, the average number of proteins on each EV was 2.85 in cancer samples and 3.40 in controls (*P* < 0.01, Fig. 2D). Overall, we identified 198 EV surface proteins in the current study, 38 of which were referred to as EV markers in the MISEV2018 guidelines [8] (Table S3). All samples showed the presence of these EV markers. In the category of transmembrane or GPI‐anchored proteins, over 75% of single EVs expressed at least one non‐tissue‐specific protein, and over 30% expressed at least one cell‐/tissue‐specific protein. (Fig. S1). The relevant content of EV characterization is also summarized in a separated file for easy reference (Table S4). The results of batch effect adjustment and normalization are shown in Figs S2 and S3, respectively.

### 3.2 DEPs between cancer and control groups

**Fig. 3:**
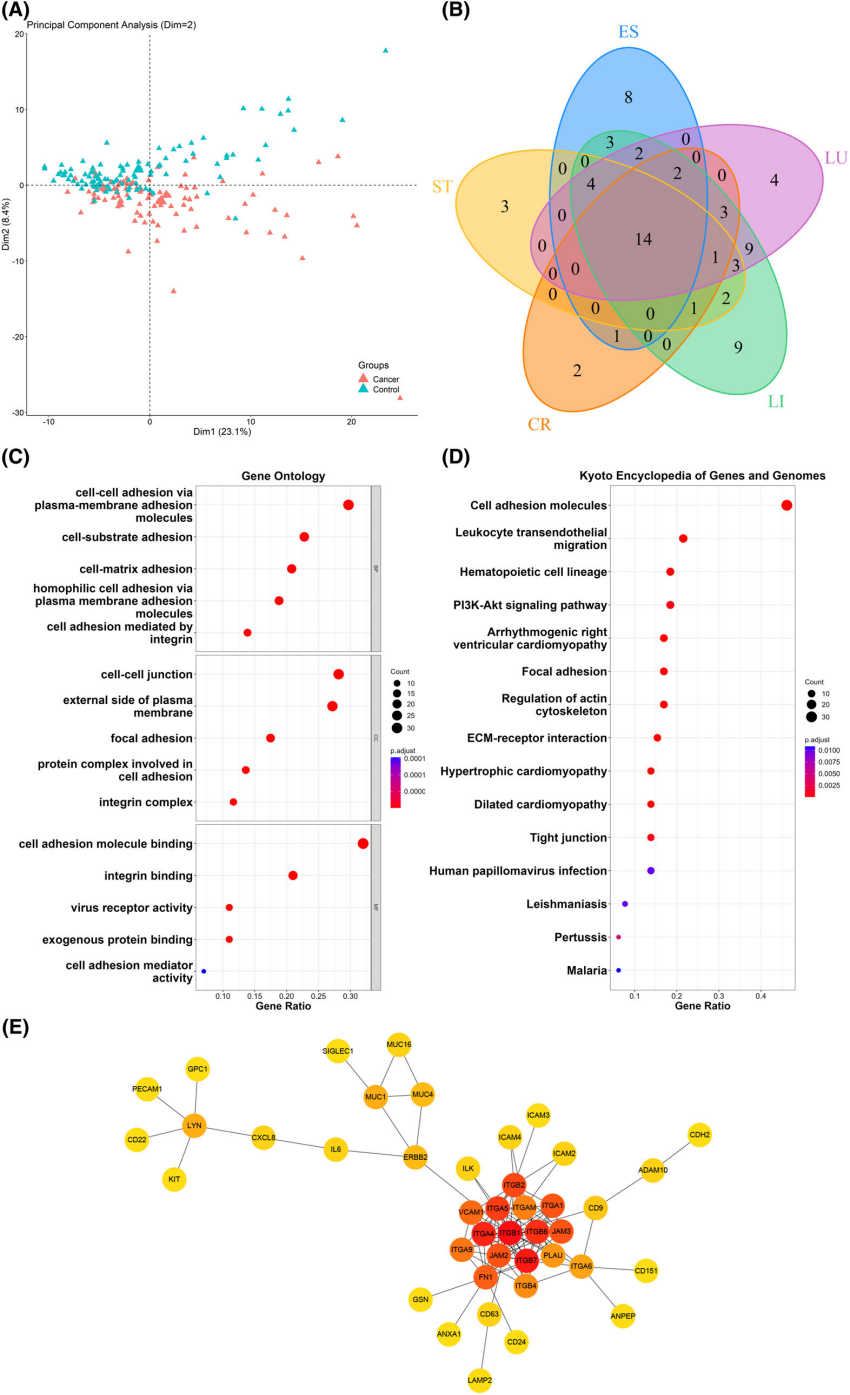
DEPs (*P*‐adj <0.05) between cancer and control groups and functional analyses. (A) Principal component analysis of EV surface proteins for pan‐cancer (*n* = 100) and control (*n* = 100) groups. (B) Venn diagram for DEPs between each cancer type (*n* = 20) and control (*n* = 100) groups. (C) GO analysis for pan‐cancer DEPs. (D) KEGG analysis for pan‐cancer DEPs. (E) Protein–protein interaction analysis for pan‐cancer DEPs.

Principal component analysis (PCA) revealed a clear separation in surface protein profiles (Fig. 3A) between the cancer and control groups. Overall, 103 DEPs of 207 PBA‐integrated proteins were differentially expressed in cancer patients versus controls (*P*‐adj <0.05), of which 36 were up‐ and 67 were downregulated (Table S5, Fig. S4A). In terms of the comparison between each type of cancer patients and controls, we detected 34 DEPs for esophageal, 28 for stomach, 24 for colorectal, 53 for liver, and 42 for lung cancer, with 14 DEPs being common among all cancer types (Fig. 3B). Table S6 and Fig. S4B–F show DEP lists and volcano plots for the five cancer types, respectively.

### 3.3 Functional enrichment and PPI analyses

All of the 103 DEPs between cancer patients and controls were then subjected to functional enrichment analysis. Considering that these proteins are localized to EV membrane, we set the 39 716 human membrane proteins in the UniProt database (subcellular location: “membrane [SL‐0162]” and organism: “Homo sapiens (Human/Man) [9606]”) as the reference protein list. GO analysis showed that most of these DEPs were enriched in cell adhesion, junction, and integrin binding (Fig. 3C). Furthermore, KEGG analysis revealed the cell adhesion molecule pathway to be the most significant pathway, involving > 45% mapped genes, followed by leukocyte transendothelial migration, hematopoietic cell lineage, and phosphatidylinositol 3‐kinase/Akt (PI3K/Akt)‐signaling pathway (Fig. 3D). PPI analysis demonstrated interactions among DEPs (Fig. 3E), suggesting that integrin subunits (e.g., ITGB1 and ITGA1), mucin family proteins (e.g., MUC1 and MUC4), and fibronectin 1 (FN1) act as hub proteins.

### 3.4 Performance of DEPs as biomarkers in cancer identification

**Fig. 4:**
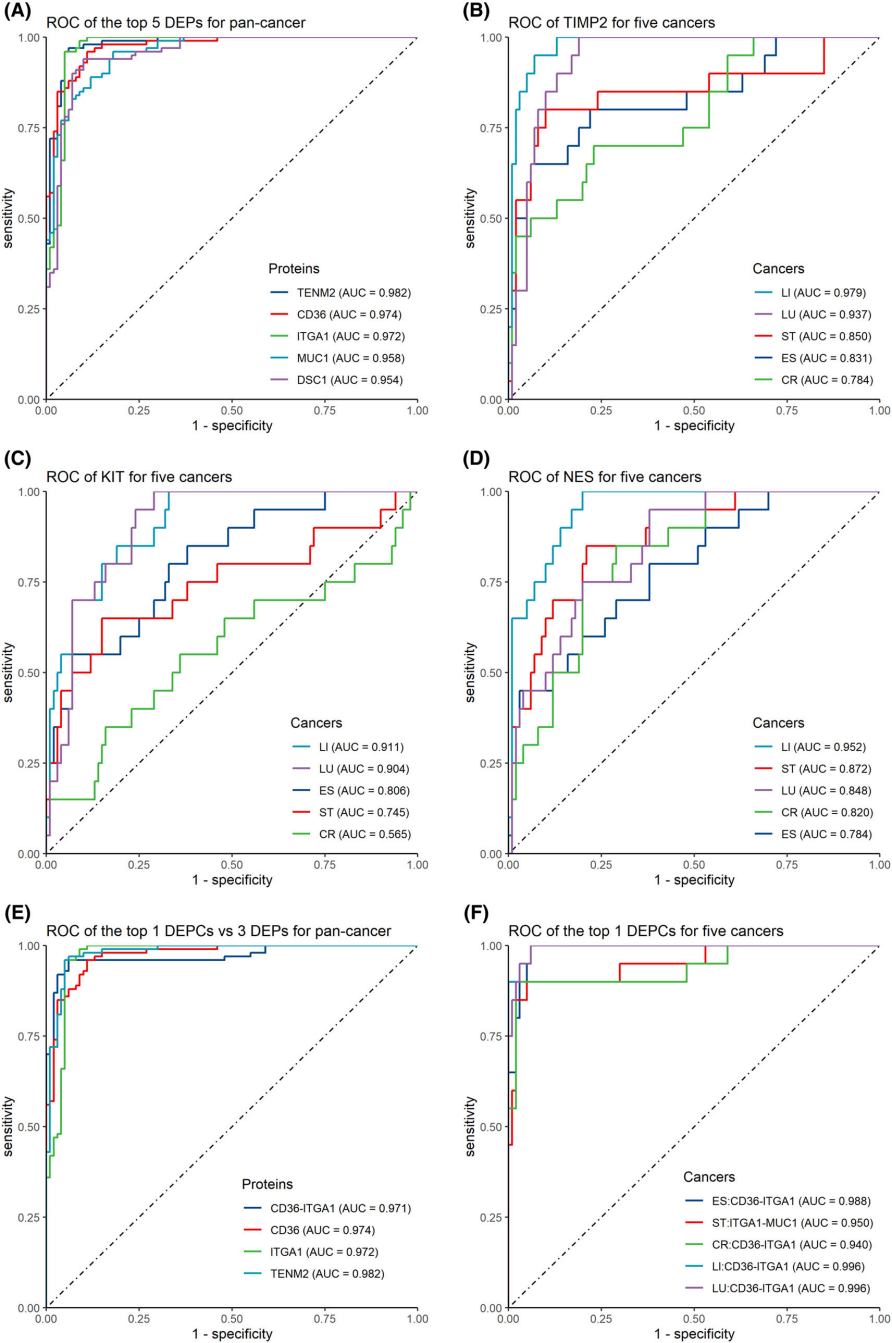
Performance of surface proteins in classifying cancer and control groups. (A) ROC of the top 5 DEPs in classifying pan‐cancer (*n* = 100) and control (*n* = 100) groups. ROC of (B) TIMP2, (C) KIT, and (D) NES in classifying each cancer type (*n* = 20) and control (*n* = 100) groups. (E) ROC curves of the top 1 DEPCs and 3 DEPs in classifying pan‐cancer (*n* = 100) and control (*n* = 100) groups. (F) ROC of the top 1 DEPCs in classifying each cancer type (*n* = 20) and control (*n* = 100) groups. DEPs and DEPCs were ranked by AUC value from highest to lowest.

In the context of pan‐cancer, the classification power of all DEPs was evaluated by ROC analysis. Overall, we identified five proteins with AUC > 0.900: teneurin transmembrane protein 2 (TENM2, AUC = 0.982, 95% CI: 0.966–0.997), CD36 (AUC = 0.974, 95% CI: 0.955–0.992), ITGA1 (AUC = 0.972, 95% CI: 0.949–0.996), MUC1 (AUC = 0.958, 95% CI: 0.934–0.982), and desmocollin 1 (DSC1, AUC = 0.954, 95% CI: 0.926–0.982; Fig. 4A). Another six proteins with AUC > 0.800, namely ANXA1, cadherin 13 (CDH13), TIMP metallopeptidase inhibitor 2 (TIMP2), nestin (NES), lymphocyte activating 3 (LAG3), and endothelial cell adhesion molecule (ESAM), could differentiate between cancer patients and controls. Table 1 shows the top 10 DEPs with the highest AUC values. Other details, such as sensitivity, specificity, and best threshold, are shown in Table S7.

**Table 1:** DEPs with top 10 AUC values for distinguish patients with pan‐cancer (*n* = 100) or each cancer type (*n* = 20) from controls (*n* = 100). DEPs were ranked by AUC value from highest to lowest. Protein names were provided in Table S2 due to limited space.

Rank	Pan‐cancer		Esophageal cancer		Stomach cancer		Colorectal cancer		Liver cancer		Lung cancer	
	Protein	AUC	Protein	AUC	Protein	AUC	Protein	AUC	Protein	AUC	Protein	AUC
1	TENM2	0.982	CD36	0.988	TENM2	0.985	TENM2	0.982	TENM2	0.988	ITGA1	0.980
2	CD36	0.974	TENM2	0.986	CD36	0.979	CD36	0.980	ITGA1	0.983	TENM2	0.968
3	ITGA1	0.972	MUC1	0.972	ITGA1	0.967	DSC1	0.966	MUC1	0.983	CD36	0.959
4	MUC1	0.958	DSC1	0.968	MUC1	0.958	ITGA1	0.964	TIMP2	0.979	TIMP2	0.937
5	DSC1	0.954	ITGA1	0.968	DSC1	0.950	MUC1	0.945	CD36	0.964	DSC1	0.937
6	ANXA1	0.897	ANXA1	0.903	ANXA1	0.912	ANXA1	0.887	NES	0.952	MUC1	0.932
7	CDH13	0.885	CDH13	0.895	CDH13	0.887	CDH13	0.877	DSC1	0.951	KIT	0.904
8	TIMP2	0.876	ESAM	0.839	NES	0.873	NES	0.820	KIT	0.911	ANXA1	0.893
9	NES	0.855	TIMP2	0.831	TIMP2	0.850	LAG3	0.786	ANXA1	0.892	CDH13	0.880
10	LAG3	0.818	LAG3	0.830	ESAM	0.821	TIMP2	0.785	CDH13	0.885	NES	0.848

For each cancer type, ROC analysis was performed using DEPs with the aim of evaluating their classification performance. Table 1 shows the top 10 DEPs with the highest AUC values. Consistent with pan‐cancer‐related findings, TENM2, ITGA1, CD36, DSC1, and MUC1 still showed good performance in terms of distinguishing between patients with each type of cancer and controls; their AUC values were > 0.900, with the highest value being 0.988 for esophageal and liver cancer. Furthermore, we observed that some proteins were significantly more effective in identifying some types of cancer than others. For example (Fig. 4B–D), TIMP2 could efficiently discriminate between controls and patients with liver cancer and lung cancer, with AUC values of 0.979 and 0.937, respectively; however, this effect was not that effective for esophageal cancer (AUC = 0.831), stomach cancer (AUC = 0.850), and colorectal cancer (AUC = 0.785). Similarly, KIT proto‐oncogene (receptor tyrosine kinase, KIT) could effectively distinguish between controls and patients with liver cancer (AUC = 0.911) and lung cancer (AUC = 0.900), but this effect was not as reliable for esophageal, stomach, and colorectal cancer (AUC <0.810). The effect of NES was reliable only for liver cancer (AUC = 0.952) than for the other four cancers (AUC < 0.850). Other results pertaining to DEPs for each cancer with AUC > 0.800 are reported in Table S8.

### 3.5 Performance of DEPCs in cancer identification

Overall, 1094 DEPCs were found between cancer patients and controls (*P*‐adj < 0.05, Table S9). For pan‐cancer identification, we performed ROC analysis to determine the frequency of EVs with all DEPCs to assess their effectiveness in distinguishing between patients with cancer and controls. AUC values of eight DEPCs reached 0.900, being less than those of DEPs; furthermore, AUC values of up to 130 DEPCs reached 0.800. CD36‐ITGA1 showed the highest AUC value, but it is notable that it showed no significant improvement in classification performance (AUC = 0.971, 95% CI: 0.948–0.994) relative to CD36 (AUC = 0.974), ITGA1 (AUC = 0.972), or TENM2 (AUC = 0.982) as DEP biomarkers (Fig. 4E). DSC1‐ITGA1 (AUC = 0.929, 95% CI: 0.894–0.965) and ITGA1‐TENM2 (AUC = 0.926, 95% CI: 0.890–0.963) ranked second and third in terms of classification performance, respectively. Table S10 shows results of protein combinations with AUC values of > 0.800.

For each cancer type and control, DEPCs (*P*‐adj <0.05, Table S11) showed no significant improvements in classification performance in comparison to DEPs. As shown in Fig. 4F, CD36‐ITGA1 was the most effective DEPC for the identification of esophageal cancer (AUC = 0.988), colorectal cancer (AUC = 0.940), liver cancer (AUC = 0.996), and lung cancer (AUC = 0.996). For stomach cancer, ITGA1‐MUC1 showed the best performance (AUC = 0.950), followed by CD36‐ITGA1 (AUC = 0.938). Of all DEPCs, the two‐by‐two combination of ITGA1, CD36, MUC1, DSC1, and TENM2 showed the most prominent classification performance. Table S12 shows results of DEPCs for each cancer type with AUC values of > 0.800.

### 3.6 Identification of EV subpopulations and comparison within EV clusters

**Fig. 5:**
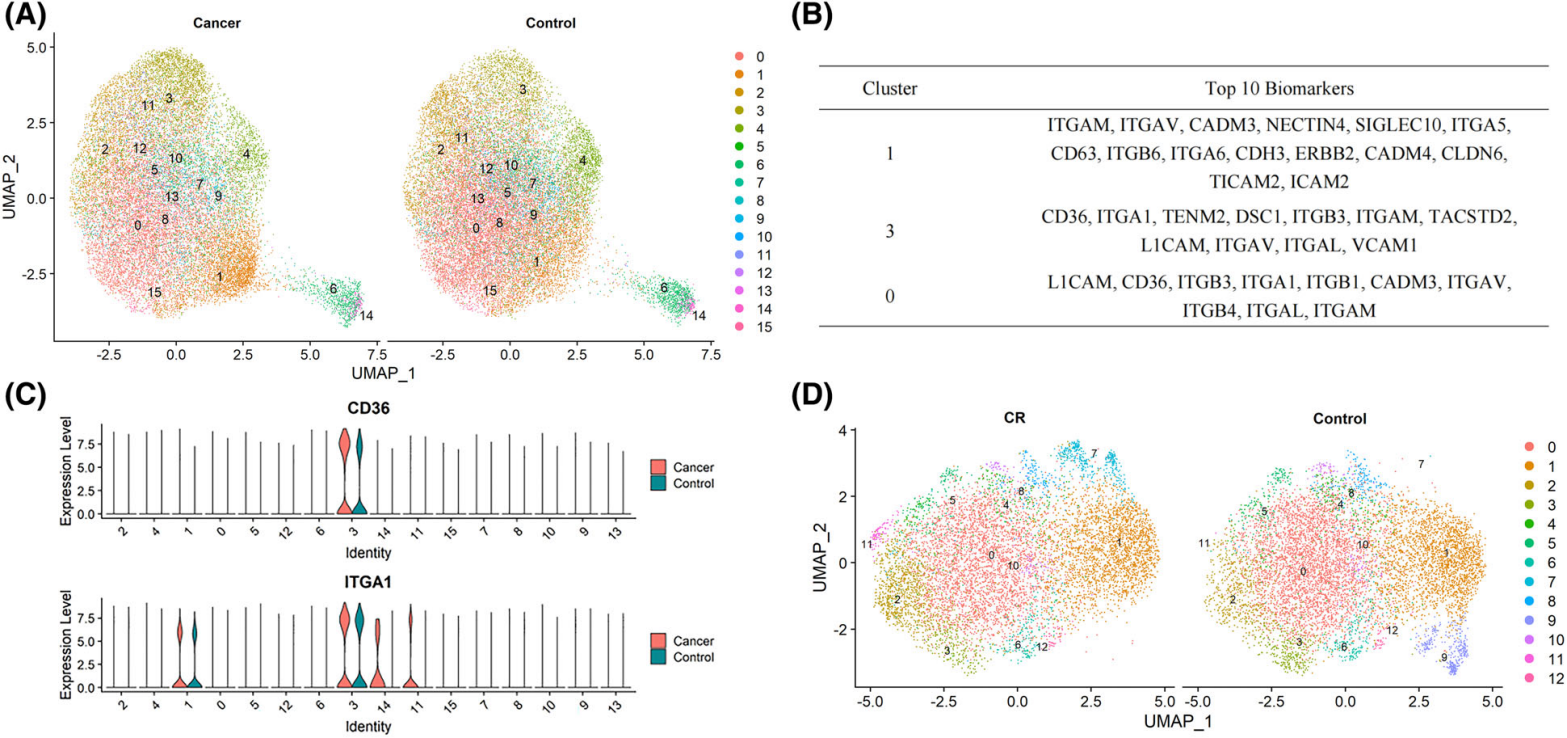
Results of EV subpopulation analyses. The down‐sampling strategy was applied to select 20 000 EVs from pan‐cancer samples (*n* = 100) and 20 000 EVs from the control samples (*n* = 100). (A) Distribution of EV subpopulations between cancer and control groups. (B) Characteristic markers of cluster 1, cluster 3, and cluster 0 in (A). (C) Distribution of CD36 and ITGA1 between cancer and control groups in all clusters. The down‐sampling strategy was also applied to select 10 000 EVs from colorectal cancer samples (*n* = 20) and 10 000 EVs from the control samples (*n* = 100). (D) Distributions of EV subpopulations between colorectal cancer and control groups.

One unique feature of the PBA approach is its ability to quantify protein expression at a single EV level, which produces a novel EV data format that is similar to single‐cell RNA‐seq data, allowing the exploration of EV population heterogeneity. Considering the large number of EVs in each sample, we applied a down‐sampling strategy and selected 20 000 EVs from the pan‐cancer group and 20 000 EVs from the control group. Seurat was applied for data visualization and EV subpopulation identification [32]. EVs were clustered into 16 subpopulations (Fig. 5A). We calculated EV composition between patients with cancer and controls in each cluster and found that cluster 1, cluster 3, and cluster 0 showed the highest intergroup variation (Table S13). Cluster 1 and cluster 3 showed a higher proportion in the cancer group, while cluster 0 showed a higher proportion in the control group. They were characterized by the protein markers presented in Fig. 5B. On searching the Human Protein Atlas database [36, 37], we found that multiple proteins, including integrin subunit alpha M (ITGAM), sialic acid‐binding Ig‐like lectin 10 (SIGLEC10), integrin subunit alpha L (ITGAL), and integrin subunit beta 3 (ITGB3), were highly expressed in immune cells and organs. ITGAM, cell adhesion molecule 3 (CADM3), and L1 cell adhesion molecule (L1CAM) tended to show a relatively high expression level in nerve cells and the nervous system (Fig. S5). In addition, we analyzed DEPs between cancer and control samples within each cluster. The cancer group showed significantly higher expression of CD36 in cluster 3 and ITGA1 in cluster 3, cluster 4, and cluster 11 (Fig. 5C). These findings were in concordance with the aforementioned results, validating DEPs from bulk EV to single EV level. For better visualization, we also divided the 20 000 EVs from the pan‐cancer group into subgroups based on the cancer types of their origins (Fig. S6).

To obtain more accurate results of subpopulations in each cancer type, we further down‐sampled 10 000 EVs from each cancer group and 10 000 EVs from the control group. The distribution of EV subpopulations and comparison between patients with colorectal cancer and controls are illustrated in Fig. 5D. We noticed that Cluster 9 was only present in control group, which was characterized by neuroligin 1 (NLGN1), protein tyrosine phosphatase receptor type J (PTPRJ), hepatitis A virus cellular receptor 2 (HAVCR2), vascular cell adhesion molecule 1 (VCAM1), and L1CAM. Biomarkers in cluster 9 such as HAVCR2 and VCAM1 are highly expressed in immune‐related cells (Fig. S5) [36, 37]. We also found that cluster 7 and cluster 11 were mainly distributed in colorectal group. The predominant biomarkers of cluster 7 were leucine‐rich repeat including G‐protein‐coupled receptor (LGR5), A disintegrin and metalloproteinase 10 (ADAM10), and integrin subunit alpha 11 (ITGA11). The major biomarkers of cluster 11 comprised integrin‐linked kinase (ILK) and lysosomal‐associated membrane protein 1 (LAMP1). Other details of the cluster‐specific proteins for the three clusters were provided in Table S14. In addition, the distribution of EV subpopulations in the other four cancers was presented in Fig. S7.

### 3.7 Classification models using EV proteins

In the pan‐cancer classification model, we included the top 10 DEPs and DEPCs in ascending order of adjusted *P*‐values (Tables S5 and S9) in the feature selection process employing stepwise bidirectional elimination, Lasso, and Random Forest algorithms. For DEPs, we individually identified five biomarkers as important variables. Notably, four proteins, TENM2, CDH13, MUC1, and ITGA1, were consistently chosen by at least two of these methods. For DEPCs, four to six features were selected by the three methods, respectively, and six DEPCs, namely CD36‐ITGA1, CD36‐DSC1, ITGA1‐MUC1, CDH13‐ITGA1, ITGA1‐TENM2, and DSC1‐ITGA1, were selected through the application of at least two different methods. The development of the two logistic regression models used the aforementioned five DEPs and six DEPCs, respectively. In the 10‐fold cross‐validation, the mean AUC value of test sets was found to be desirable irrespective of using data of DEPs (AUC = 0.981, 95% CI: 0.943–0.999) or DEPCs (AUC = 0.965, 95% CI: 0.903–0.999). Then, we added smoking status, drinking status, and family history of cancer into the present model and found no significant change in model performance. We also performed similar methods to construct models for distinguishing gastrointestinal cancer patients (esophageal, stomach, and colorectal cancer, *n* = 60) from controls (*n* = 100), and the AUC values were 0.985 (0.952, 0.999) using DEPs and 0.965 (0.891, 0.999) using DEPCs. More details of the classification models are provided in Table 2.

**Table 2:** The classification model for distinguishing cancer patients from controls using EV proteins. The feature selection and model construction process were conducted in the discovery dataset. The protein biomarkers selected by at least two algorithms were used for the model construction. The models were developed by logistic regression and evaluated by 10‐fold cross‐validation in the discovery dataset and the AUC value was the average result of 10 test sets. The validation dataset was only used for model evaluation. “Bold” represents the overlapping biomarkers. Protein names were provided in Table S2 due to limited space.

Pan‐cancer vs. Control	Selected variable	Logistic model	Discovery dataset (*n* = 200, 100 vs. 100)		Validation dataset (*n* = 28, 14 vs. 14)
			AUC (95% CI)	AUC (95% CI)^a^	AUC (95% CI)
DEPs					
Stepwise	**TENM2**, **MUC1**, DSC1, **CDH13**, LAG3	TENM2, MUC1, CDH13, ITGA1	0.981 (0.943, 0.999)	0.983 (0.949, 0.999)	0.786 (0.607, 0.801)
LASSO	**TENM2**, **ITGA1**, **CDH13**, NES, ANXA1				
Random forest	CD36, **ITGA1**, TENM2, **MUC1**, **CDH13**				
DEPCs					
Stepwise	**CD36‐ITGA1**, **CD36‐DSC1**, **ITGA1‐MUC1**, **CDH13‐ITGA1**	CD36‐ITGA1, CD36‐DSC1, ITGA1‐MUC1, CDH13‐ITGA, ITGA1‐TENM2, DSC1‐ITGA1	0.965 (0.903, 0.999)	0.967 (0.909, 0.999)	0.622 (0.403, 0.627)
LASSO	CD36‐EPCAM, **CDH13‐ITGA1**, **ITGA1‐MUC1**, **ITGA1‐TENM2**, **DSC1‐ITGA1**, **CD36‐ITGA1**				
Random forest	**CD36‐ITGA1**, **DSC1‐ITGA1**, **CD36‐DSC1**, **ITGA1‐MUC1**, **ITGA1‐TENM2**				

Gastrointestinal Cancer vs. Control	Selected variable	Logistic model	Discovery dataset (*n* = 160, 60 vs. 100)		Validation dataset (*n* = 24, 10 vs. 14)
			AUC (95% CI)	AUC (95% CI)^a^	AUC (95% CI)
DEPs					
Stepwise	**TENM2**, **CD36**, MUC1, **CDH13**, ESAM	TENM2, CD36, CDH13	0.985 (0.952, 0.999)	0.990 (0.965, 0.999)	0.807 (0.607, 0.814)
LASSO	**TENM2**, **CDH13**, NES				
Random forest	ITGA1, **CD36**, **TENM2**, DSC1, **CDH13**				
DEPCs					
Stepwise	**CD36‐ITGA1**, **DSC1‐ITGA1**, **ITGA1‐TENM2**, **ITGA1‐MUC1**, **CD36‐DSC1**, **CD36‐VCAM1**, ITGA1‐TIMP2	CD36‐ITGA1, DSC1‐ITGA1, ITGA1‐TENM2, ITGA1‐MUC, CD36‐DSC1, CD36‐VCAM1	0.965 (0.891, 0.999)	0.970 (0.908, 0.999)	0.714 (0.486, 0.721)
LASSO	CD36‐EPCAM, **ITGA1‐MUC1**, **ITGA1‐TENM2**, **DSC1‐ITGA1**, CDH13‐ITGA1, **CD36‐VCAM1**, CD36‐ITGAV				
Random forest	**CD36‐ITGA1**, **ITGA1‐MUC1**, **DSC1‐ITGA1**, **CD36‐DSC1**, **ITGA1‐TENM2**				

^a^ Adjusting for epidemiological factors: smoking, drinking, and family history of cancer.

For cancer‐type‐specific classification, we identified DEPs and DEPCs for each cancer type against the other four cancers (Tables S15 and S16) and ranked the results using adjusted *P*‐values. To generate the classification models, we filtrated the top 10 DEPs and DEPCs by bidirectional elimination to select biomarkers. The remaining DEPs and DEPCs were used to build the logistic regression models for distinguishing patients with one cancer from the other four cancers, respectively. In the leave‐one‐out cross‐validation, we observed that the model constructed using DEPs had an accuracy of 78–84% in distinguishing each cancer from the other four, while that constructed using DEPCs had an accuracy of 85–92%. Table 3 summarizes the results of these classification models.

**Table 3:** The classification model for distinguishing patients with one cancer (*n* = 20) from the other four (*n* = 80) using EV proteins. The models were developed by logistic regression and evaluated by leave‐one‐out cross‐validation, which included the DEPs or DEPCs filtrated by bidirectional elimination. Protein names were provided in Table S2 due to limited space. Accuracy: proportion of samples with correct predictions.

Model	DEPs		DEPCs	
	Variable	Accuracy	Variable	Accuracy
ES/Non‐ES	PDCD1, CD24, TMEM204, CD81	78%	CD20‐MUC1, CXCL16‐DSC1, MUC4‐PCDHA1, CLEC1B‐THY1, DSCAM‐RETN	85%
ST/Non‐ST	PECAM1, JAM2, CEACAM3, CSPG4, TIM3	84%	CLDN4‐CLEC1B, CD26‐PROM1, CLDN17‐SIGLEC1, CLDN12‐GPC1, RETN‐ULBP3, GSN‐TIMP2, GPC1‐TICAM2	91%
CR/Non‐CR	GSN, KIT, MICA, CD274, NTRK3, CLDN19	83%	CD44‐TENM2, CCR6‐CLDN4, CSPG4‐DSC1, NECTIN1‐PLAU, ADAM10‐SIGLEC11, MUC16‐ULBP3, DSC1‐EMCN	91%
LI/Non‐LI	TIMP2, GSN, CLEC1B, MUC1, CD81	83%	CD26‐PCDH17, CDH3‐CLEC1B, CD63‐NGFR, ICAM3‐ITGB1, ITGB8‐SIGLEC10, CEACAM5‐MICA, CEACAM3‐MICA	92%
LU/Non‐LU	GSN, CD9, CDH6, CDH1	81%	SELE‐TENM4, CDH1‐PCDHGC3, CLEC2A‐MUC16, ANXA1‐NGFR, PTPRJ‐TIMP2, MUC4‐PCDH15, CDH2‐CDON	86%

### 3.8 Classification model performance in the validation dataset

We evaluated the classification performance of the constructed model in the validation dataset (*n* = 28). As Table 2 shows, given the small number of samples, we still achieved an AUC value of 0.786 (95% CI: 0.607–0.801) using selected DEPs to distinguish cancer patients from controls (*P* < 0.05). The post hoc analysis shows that the DEPs achieved a statistical power at 0.799 (Fig. S8). The AUC using DEPCs was lower than DEPs at 0.622, which was in agreement with that DEPs had better classification performance than DEPCs in the discovery dataset. Of the 14 cancer samples, 10 of them belonged to gastrointestinal cancer (stomach, colorectal, and esophageal cancer) and the other 4 were liver and lung cancer. Interestingly, when distinguishing gastrointestinal cancer from control samples, the AUC increased to 0.807 for DEPs and 0.714 for DEPCs.

## 4 Discussion

Although a few studies have reported EV proteins to be biomarkers for cancer detection, most of them only involved one type of cancer, limiting the possibility of exploring commonalities and specificities among different cancers. In this study, we assessed five types of cancers as a group to profile plasma EV surface proteins using PBA, which led to the identification of 103 DEPs. Many of these DEPs were found to be associated with cancer development (e.g., cell adhesion, leukocyte migration, and PI3K/Akt‐signaling pathway) [38, 39, 40]. TENM2, CD36, ITGA1, MUC1, and DSC1 were identified to be the most promising biomarkers in terms of classification performance and significance level; their expression levels were significantly upregulated across all five cancers. The main functions of these proteins are cell adhesion and signal transduction. Many studies have suggested that CD36 plays a vital role in fatty acid metabolism, which is crucial to energy utilization by tumor cells and for differentiation and activation of immune cells [41, 42]. As a member of integrin receptors, a previous study reported ITGA1 to be a new diagnostic biomarker as its expression was upregulated in pancreatic ductal adenocarcinoma tissues and serum of patients with colorectal cancer [43, 44]. MUC1 overexpression is evidently associated with carcinogenesis [45]. MUC1 participates in regulating programmed cell death, mediates the resistance to apoptosis, and impacts the elimination of damaged cells [46]. With regard to EV proteins, an earlier study showed that EVs‐containing CD36 can be endocytosed by hepatocytes, inducing lipid accumulation and inflammation development, consequently promoting nonalcoholic fatty liver disease development [47]. Consistent with the five types of cancers assessed in this study, MUC1 of EVs was also identified as the candidate biomarker in pancreatic juice of patients with pancreatic cancer and in the plasma of those with breast cancer [48, 49]. However, only a few studies have explored TENM2, ITGA1, and DSC1 in EVs. To our knowledge, this can be partly explained by differences among diverse detection methods. In this study, all proteins detected using the PBA approach were located on EV surface and may not be detectable by other methods.

In comparison to traditional methods, such as western blotting and ELISA, one of the advantages of PBA is its ability to produce a wealth of protein information at a single EV level, which is similar to single‐cell RNA‐seq data. Apart from bulk protein expression data of all EVs, single EV data help in revealing the heterogeneity of each EV and EV subpopulation. It is worth noting that the average number of proteins detected on each EV was around three, which is inconsistent with the recognized viewpoints of the abundant proteins carried by EVs. Considering the small volume of EVs and the limited number of proteins in the PBA panel, while a typical cell usually expresses proteins from several thousands of genes, it would be reasonable if we detected only about 2–3 proteins per EV. Furthermore, the point of “EVs contain abundant proteins” comes from EV bulk analysis without the consideration of EV heterogeneity; thus, it may be reasonable to suggest that only several proteins are expressed on average in each EV [50]. Besides, steric impediments could occur in antibody‐based techniques. However, we assume that this detection efficiency is consistent across all samples, ensuring the reliability of significant differences between disease and control samples. The single EV data were fully utilized for the exploratory analysis of EVs with specific protein combinations, namely the co‐expression of EV surface proteins and EV subpopulation classification. For DEPCs, we found that the optimal classification effect was provided by the two‐by‐two combination of CD36, ITGA1, MUC1, TENM2, and DSC1 for both pan‐cancer and individual cancer types. The best performance of DEPCs was not significantly better than that of DEPs as individual protein biomarkers, but we noticed the improvement in cancer‐type‐specific identification when using DEPCs, detailed in next paragraph of classifier development.

Based on the protein profile of each EV, we divided EVs into different subpopulations using clustering algorithm. Some DEPs, such as CD36 and ITGA1, were detected again in specific clusters, indicating that findings obtained using single and bulk EV protein data were mutually validated. We also found the proportion of some clusters, comprising several proteins that were proven to be highly expressed in immune cells [36, 37], such as ITGAM, SIGLEC10, ITGAL, and ITGB3, was significantly different between all patients with cancer and controls, suggesting quantitative changes in immune‐related subpopulations during cancer progression. For colorectal cancer, we detected that cluster 9 was exclusively present in the control group, with a significant upregulation of NLGN1, PTPRJ, and HAVCR2. HAVCR2 was reported to be involved in regulating antitumor immunity [51], and PTPRJ was known for its antiproliferative role and inhibits the tumorigenicity of colorectal cell lines in a xenograft tumor model [52]. We infer that the absence of cluster 9 in cancer patients promotes tumorigenesis. The proportion of cluster 7 characterized by LRG5 was significantly higher in colorectal cancer group. LRG5 plays a crucial role in the formation and maintenance of adult intestinal stem cells during postembryonic development [45]. A previous study reported that human LGR5+ colorectal cancer cells serve as cancer stem cells in growing cancer tissues [53]. It has been proven that selective LGR5+ cell ablation in the mouse model can suppress primary tumor growth [54]. In addition, cluster 11 also exhibited high expression in colorectal cancer group, and its main marker LAMP1 was identified as a significant marker in the formation and maintenance of endolysosomes within colorectal cancer stem cells [55]. The potential discovery of colorectal cancer stem cell‐related EV subpopulations may offer new insights into targeting colorectal cancer stem cells as a novel therapeutic approach [56]. Further exploration and validation are needed to elucidate the functions of these subpopulations.

As Ferguson et al. [50] summarized in a review, single EV analysis can shed light on circulating tumor‐derived EVs and determining their organ of origin, but most of the existing studies only involved later‐stage cancers, single‐marker studies, or a subset of single EVs utilizing immunoaffinity capture. In this study, we utilized the multiplexed assay to detect 207 protein biomarkers simultaneously, allowing the possibility of high‐dimensional data analysis and interpretation. One of the advantages of single EV analysis is improving the sensitivity of detecting minor cancer signals at an early stage, instead of being diluted by background signals in bulk analysis. This has not been demonstrated in our current stage of the study due to the lack of cancer stage information, so we are unable to observe the trend effect of the EV surface protein expression level among different cancer stages. Further studies, involving cancer patients prospectively recruited before the clinical diagnosis or at different stages, should be conducted to provide evidence for the biomarkers in detecting cancer in advance [57].

To further discover the potential of pan‐cancer‐ and cancer‐type‐specific identification, we attempted to build several classification models based on EV surface proteins. In general, pan‐caner identification was promising, while cancer‐type‐specific classifications were challenging, meaning that most patients with cancer could be distinguished from controls but it was tough to trace the origin organ of cancer. Hoshino et al. [6] performed comprehensive proteomic profiling of EVs from tissues and plasma obtained from patients with melanoma and colorectal, pancreatic, and lung cancer; these four cancer types could be completely distinguished based on protein expression levels of tissue or plasma EVs. On one hand, the utilized PBA panel can only detect 207 proteins, which may limit the discovery of DEPs. On the other hand, esophageal, stomach, colorectal, and liver cancer are all digestive system cancers and thus involve similar physiopathological processes [58, 59, 60]. This can possibly make differentiation among cancer types difficult. Furthermore, in this study, we observed that DEPCs showed better performance in cancer‐type‐specific identification. As a novel format of protein profiling, protein combination patterns can achieve approximately 5–10% more accuracy than individual protein expression, indicating the potential of protein combinations in the classification of cancer types. In addition, the constructed model for distinguishing cancer patients from controls still achieved an AUC value of 0.786 with a statistical power of approximately 80% using selected DEPs in the validation dataset. We consider it still confirms the effectiveness of the model in cancer identification to a certain extent, despite the limited sample size. The AUC using DEPCs was lower than DEPs, which was in agreement that DEPs had better classification power than DEPCs in the discovery dataset. We infer that DEPCs represent the combination of EV proteins, which could be less frequent than DEPs and difficult to quantify due to biological and technical limitations, thus leading to sparse data on DEPCs. Therefore, we still need more data to validate the effect of DEPCs. It is worth noting that the model performance improved when distinguishing gastrointestinal cancer from control samples. We speculate that there is cancer‐specific heterogeneity impairing the classification accuracy, it would be beneficial to identify and adjust such heterogeneity in EV protein data. We will consider this as a major point in our following study.

In comparison to EVs, circulating free DNA (cfDNA)‐based liquid biopsy approaches have achieved favorable performance in pan‐cancer classifying such as “Galleri”, “PanSeer”, and “CancerSEEK” [24, 61, 62]. As a cfDNA methylation‐based method, “Galleri” could identify over 50 cancer types across stages in a case–control study, and an independent validation study was conducted to confirm its accuracy for cancer signal detection as well as cancer signal origin [62, 63]. “PanSeer” was designed as a population‐based study, involving cancer patients and prospectively collected prediagnostic participants, and it demonstrated the ability to detect cancer in 80% of cancer patients and 95% of asymptomatic individuals who were diagnosed with the five cancers in our study within 4 years [24]. Lennon et al. [64] performed a prospective, interventional study in a health system to evaluate the feasibility of “CancerSEEK” blood test combined with PET‐CT, which proved its efficacy in routine clinical care to screen pan‐cancer. To summarize, the clinical application of liquid biopsy approaches must be based on high‐level epidemiological evidence. Just as in our study, most EV biomarker research for pan‐cancer is still at the stage of accumulating retrospective evidence, suggesting the long way to real‐world application. In addition, various machine learning methods are involved in the development of the aforementioned cancer classifiers, implying the great potential of combining EV analysis and machine learning in cancer liquid biopsy [5].

Our study has several strengths. First, we identified potential biomarkers shared by pan‐cancer using retrospectively stored samples in a case–control design, generating evidence for a subsequent study involving a population‐based cohort, with plasma samples collected 1 to 5 years before the clinical diagnosis of cancer. Our ultimate goal is to verify the ability of EV proteins to detect cancer before conventional clinical diagnosis. Second, we analyzed data at a single EV level to interpret the heterogeneity of EVs, producing high‐throughput and novel data for EV research. Third, although it is challenging to distinguish different cancers, we found DEPCs to be advantageous in cancer‐type‐specific classification. This study also has several limitations. The first is the limited number of proteins that were profiled in the current PBA panel, restricting the discovery of some potential biomarkers, especially cancer‐type‐specific proteins. Second, the study only used one technique and bioinformatic analysis to obtain the results, lacking confirmation by another technique. The sample size of the validation dataset has also limited the validation performance, indicating the necessity to construct a larger cohort to validate our findings. Third, we cannot observe the trend effect of the EV surface protein expression level among different cancer stages due to the lack of tumor, node, and metastasis information. Finally, we did not investigate the mechanisms of specific proteins in pathological processes.

## 5 Conclusions

To summarize, as one of the few pan‐cancer studies, we found and validated several potential EV surface protein biomarkers for diagnosing and screening five cancers. Cancer‐type‐specific identification remains difficult but data at a single EV level are expected to improve performance. Our findings provide epidemiological evidence for the value of EV proteins both in pan‐cancer and cancer‐type‐specific identification and implicate their potential functional roles in cancer physiopathology, laying the foundation for the future development of a non‐invasive blood assay for cancer detection and screening. To promote translational application, further prospective studies involving asymptomatic or preclinical participants from the general population are warranted to verify our results.

## Conflict of interest

Di Wu is a shareholder of Vesicode AB.

## Author contributions

YM and WD: formal analysis, visualization, and writing–original draft; HY, DZ, RZ, and PZ: data curation; JX and ZY: data curation and investigation; TZ, YJ, and KX: methodology and supervision; DW: methodology, resources, and supervision; YC: methodology, project administration, resources, and writing–review & editing; CS and XC: funding acquisition, methodology, project administration, and writing–review & editing. All authors read and approved the final manuscript.

### Peer review

The peer review history for this article is available at https://www.webofscience.com/api/gateway/wos/peer‐review/10.1002/1878‐0261.13586.

## Supporting information


**Fig. S1** Percentage of total EVs expressing at least one of the transmembrane or GPI‐anchored proteins in this study.
**Fig. S2.** Batch effect adjustment by ComBat‐seq package.
**Fig. S3.** Normalization of EV protein expression data by trimmed mean of M‐values algorithm.
**Fig. S4.** Volcano plots of DEPs (*P*‐adj < 0.05).
**Fig. S5.** Protein expression in tissues based on the Human Protein Atlas database.
**Fig. S6.** EV subpopulation distribution among five cancers and control.
**Fig. S7.** EV subpopulation distribution between each cancer and control.
**Fig. S8.** Post hoc analysis of power calculation for the classification model performance in the validation dataset, using PASS v15.
**Table S1.** Demographic and epidemiological characteristics of all cancer patients and controls in this study.
**Table S2.** List of 207 proteins on PBA panel.
**Table S3.** List of 198 proteins detected in this study and those referred to as EV markers in the MISEV2018 guidelines.
**Table S4.** MISEV2018‐based checklist for EV characterization.
**Table S5.** DEPs between pan‐cancer (*n* = 100) and control (*n* = 100).
**Table S6.** DEPs between each cancer (*n* = 20) and control (*n* = 100).
**Table S7.** Results of ROC analysis of DEPs (AUC > 0.800) for pan‐cancer.
**Table S8.** Results of ROC analysis of DEPs (AUC > 0.800) for each cancer.
**Table S9.** DEPCs between pan‐cancer (*n* = 100) and control (*n* = 100).
**Table S10.** Results of ROC analysis of DEPCs (AUC > 0.800) for pan‐cancer.
**Table S11.** DEPCs between each cancer (*n* = 20) and control (*n* = 100).
**Table S12.** Results of ROC analysis of DEPCs (AUC > 0.800) for each cancer.
**Table S13.** Binomial test for each EV subpopulation distribution between pan‐cancer (*n* = 100) and control (*n* = 100).
**Table S14.** Cluster‐specific proteins in clusters 7, 9, and 11 between colorectal cancer and control samples (*P*‐adj < 0.05 and avg.log2FC > 0).
**Table S15.** Adjusted *P*‐values of DEPs for each cancer (*n* = 20) against the other four cancers (*n* = 80).
**Table S16.** Adjusted *P*‐values of DEPCs for each cancer (*n* = 20) against the other four cancers (*n* = 80).

## Data Availability

Raw data generated in this study are not publicly available for privacy reasons. However, processed data can be accessed upon reasonable request from the corresponding author and the approval of the Human Ethics Committee of Fudan University Taizhou Institute of Health Sciences.
