# Supplementary material for: Single extracellular vesicle surface protein‐based blood assay identifies potential biomarkers for detection and screening of five cancers
Source: Mol Oncol. 2024 Jan 9;18(3):743–61. doi: 10.1002/1878-0261.13586 (PMC10920081; doi:10.1002/1878-0261.13586)
Supplement: Supplementary file 1 — Fig. S1 Percentage of total EVs expressing at least one of the transmembrane or GPI‐anchored proteins in this study. Fig. S2. Batch effect adjustment by ComBat‐seq package. Fig. S3. Normalization of EV protein expression data by trimmed mean of M‐values algorithm. Fig. S4. Volcano plots of DEPs (P‐adj < 0.05). Fig. S5. Protein expression in tissues based on the Human Protein Atlas database. Fig. S6. EV subpopulation distribution among five cancers and control. Fig. S7. EV subpopulation distribution between each cancer and control. Fig. S8. Post hoc analysis of power calculation for the classification model performance in the validation dataset, using PASS v15. Table S1. Demographic and epidemiological characteristics of all cancer patients and controls in this study. Table S2. List of 207 proteins on PBA panel. Table S3. List of 198 proteins detected in this study and those referred to as EV markers in the MISEV2018 guidelines. Table S4. MISEV2018‐based checklist for EV characterization. Table S5. DEPs between pan‐cancer (n = 100) and control (n = 100). Table S6. DEPs between each cancer (n = 20) and control (n = 100). Table S7. Results of ROC analysis of DEPs (AUC > 0.800) for pan‐cancer. Table S8. Results of ROC analysis of DEPs (AUC > 0.800) for each cancer. Table S9. DEPCs between pan‐cancer (n = 100) and control (n = 100). Table S10. Results of ROC analysis of DEPCs (AUC > 0.800) for pan‐cancer. Table S11. DEPCs between each cancer (n = 20) and control (n = 100). Table S12. Results of ROC analysis of DEPCs (AUC > 0.800) for each cancer. Table S13. Binomial test for each EV subpopulation distribution between pan‐cancer (n = 100) and control (n = 100). Table S14. Cluster‐specific proteins in clusters 7, 9, and 11 between colorectal cancer and control samples (P‐adj < 0.05 and avg.log2FC > 0). Table S15. Adjusted P‐values of DEPs for each cancer (n = 20) against the other four cancers (n = 80). Table S16. Adjusted P‐values of DEPCs for each cancer (n = 20) against [file MOL2-18-743-s001.zip › Full legends of supporting information.docx]

**Full legends of supporting information**

**Supplementary Figures:**

**Supplementary Fig. S1 Percentage of total EVs expressing at least one of the transmembrane or GPI-anchored proteins in this study.** This category of EV marker consists of non-tissue-specific and cell/tissue-specific proteins as outlined in the MISEV2018 guidelines, including ADAM10, CD63, CD81, CD9, EPCAM, ERBB2, HLA-A, HLA-DRA, ITGA1, ITGA11, ITGA2, ITGA2B, ITGA3, ITGA4, ITGA4B7, ITGA5, ITGA6, ITGA8, ITGA9, ITGAL, ITGAM, ITGAV, ITGAX, ITGB1, ITGB2, ITGB3, ITGB4, ITGB5, ITGB6, ITGB7, ITGB8, LAMP1, LAMP2, NT5E, PECAM1, THY1. EVs: extracellular vesicles; GPI: glycosyl-phosphatidyl-inositol; MISEV2018: minimal information for studies of extracellular vesicles 2018.

**Supplementary Fig. S2 Batch effect adjustment by ComBat-seq package.** PCA of plasma samples assayed on three batches before and after adjustment. PCA: principal component analysis; PBS: phosphate buffered saline; PC: positive control.

**Supplementary Fig. S3 Normalization of EV protein expression data by trimmed mean of M-values algorithm.** Comparison of EV protein expression level between cancer (*n* = 100) and control (*n* = 100) groups before and after normalization. EV: extracellular vesicle.

**Supplementary Fig. S4 Volcano plots of DEPs (p-adj < 0.05)** between (A) pan-cancer (*n* = 100) and control (*n* = 100) groups. (B) ES and control groups. (C) ST and control groups. (D) CR and control groups. (E) LI and control groups. (F) LU and control groups. B–F involves one cancer (*n* = 20) and control (*n* = 100) samples. DEPs: differentially expressed proteins; ES: esophageal cancer; ST: stomach cancer; CR: colorectal cancer; LI: liver cancer; LU: lung cancer

**Supplementary Fig. S5 Protein expression in tissues based on the Human Protein Atlas database.** Images available from v22.0.proteinatlas.org. The Human Protein Atlas database allow using images downloaded from the site when cited properly.

**Supplementary Fig. S6 EV subpopulation distribution among five cancers and control.** (A) A down-sampling strategy was applied to select 20,000 EVs from the cancer group (*n* = 100) and 20,000 EVs from the control group (*n* = 100), and (B) EVs from the cancer group were further classified by cancer type. EV: extracellular vesicle; ES: esophageal cancer; ST: stomach cancer; CR: colorectal cancer; LI: liver cancer; LU: lung cancer

**Supplementary Fig. S7 EV subpopulation distribution between each cancer and control.** A down-sampling strategy was applied to select 10,000 EVs from each cancer group (*n* = 20) and 10,000 EVs from the control group (*n* = 100), respectively. (A) ES. (B) ST. (C) LI. (D) LU. EV: extracellular vesicle; ES: esophageal cancer; ST: stomach cancer; CR: colorectal cancer; LI: liver cancer; LU: lung cancer

**Supplementary Fig. S8** **Post-hoc analysis of power calculation for the classification model performance in the validation dataset, using PASS v15.** Power is the probability of rejecting a false null hypothesis; N+ and N- are the number of items sampled from each population; N is the total sample size, N+ + N-; AUC0' and AUC1' are the adjusted areas under the ROC curve for the null and alternative hypotheses, respectively; Diff' is AUC1 - AUC0. This is the adjusted difference to be detected; AUC0 and AUC1 are the actual areas under the ROC curve for the null and alternative hypotheses, respectively; Diff is AUC1 - AUC0; This is the difference to be detected; Alpha is the probability of rejecting a true null hypothesis; FPR1, FPR2 are the lower and upper bounds on the false positive rates; B is the ratio of the standard deviations of the negative and positive groups.

**Supplementary Tables:**

**Supplementary Table S1. Demographic and epidemiological characteristics of all cancer patients and controls in this study.** *P*-value calculation: Categorical variables were analyzed using Fisher's exact test; continuous variables are analyzed using Wilcoxon rank-sum test. * *P* < 0.05.

**Supplementary Table S2. List of 207 proteins on PBA panel.** PBA: proximity-dependent barcoding assay.

**Supplementary Table S3. List of 198 proteins detected in this study and those referred to as EV markers in the MISEV2018 guidelines.** EV markers listed in the MISEV2018 guidelines are highlighted in blue; MISEV2018: minimal information for studies of extracellular vesicles 2018.

**Supplementary Table S4.** **MISEV2018-based checklist for EV characterization.** Reference: Théry C, Witwer KW, Aikawa E, et al. Minimal information for studies of extracellular vesicles 2018 (MISEV2018): a position statement of the International Society for Extracellular Vesicles and update of the MISEV2014 guidelines. J Extracell Vesicles. 2018;7(1):1535750. MISEV2018: minimal information for studies of extracellular vesicles 2018; EV: extracellular vesicle; PBA: proximity-dependent barcoding assay; GPI: glycosyl-phosphatidyl-inositol.

**Supplementary Table S5.** **DEPs between pan-cancer (*n* = 100) and control (*n* = 100).** DEPs: differentially expressed proteins; log2FC: log2(fold change).

**Supplementary Table S6.** **DEPs between each cancer (*n* = 20) and control (*n* = 100).** DEPs: differentially expressed proteins; log2FC: log2(fold change); ES: esophageal cancer; ST: stomach cancer; CR: colorectal cancer; LI: liver cancer; LU: lung cancer.

**Supplementary Table S7. Results of ROC analysis of DEPs (AUC > 0.800) for pan-cancer.** ROC: receiver operating characteristic; DEPs: differentially expressed proteins; AUC: area under curve; PPV: positive predictive value; NPV: negative predictive

**Supplementary Table S8. Results of ROC analysis of DEPs (AUC > 0.800) for each cancer.** ROC: receiver operating characteristic; DEPs: differentially expressed proteins; AUC: area under curve; TPR: true positive rate; FPR: false positive rate; PPV: positive predictive value; NPV: negative predictive; ES: esophageal cancer; ST: stomach cancer; CR: colorectal cancer; LI: liver cancer; LU: lung cancer.

**Supplementary Table S9.** **DEPCs between pan-cancer (*n* = 100) and control (*n* =100).** DEPCs: differentially expressed protein combinations; log2FC: log2(fold change).

**Supplementary Table S10. Results of ROC analysis of DEPCs (AUC > 0.800) for pan-cancer.** ROC: receiver operating characteristic; DEPCs: differentially expressed protein combinations; AUC: area under curve; TPR: true positive rate; FPR: false positive rate.

**Supplementary Table S11.** **DEPCs between each cancer (*n* = 20) and control (*n* =100).** DEPCs: differentially expressed protein combinations; log2FC: log2(fold change); ES: esophageal cancer; ST: stomach cancer; CR: colorectal cancer; LI: liver cancer; LU: lung cancer.

**Supplementary Table S12.** **Results of ROC analysis of DEPCs (AUC > 0.800) for each cancer.** ROC: receiver operating characteristic; DEPCs: differentially expressed protein combinations; AUC: area under curve; TPR: true positive rate; FPR: false positive rate; ES: esophageal cancer; ST: stomach cancer; CR: colorectal cancer; LI: liver cancer; LU: lung cancer.

**Supplementary Table S13.** **Binomial test for each EV subpopulation distribution between pan-cancer (*n* = 100) and control (*n* = 100).** EV: extracellular vesicle.

**Supplementary Table S14.** **Cluster-specific proteins in cluster 7, 9 and 11 between colorectal cancer and control samples (P.adjust < 0.05 and avg.log2FC >0).** avg. log2FC: log2(fold change) in average.

**Supplementary Table S15.** **Adjusted *P*-values of DEPs for each cancer (n = 20) against the other four cancers (n = 80).** DEPs: differentially expressed proteins; ES: esophageal cancer; ST: stomach cancer; CR: colorectal cancer; LI: liver cancer; LU: lung cancer.

**Supplementary Table S16.** **Adjusted *P*-values of DEPCs for each cancer (n = 20) against the other four cancers (n = 80).** DEPCs: differentially expressed protein combinations; ES: esophageal cancer; ST: stomach cancer; CR: colorectal cancer; LI: liver cancer; LU: lung cancer.
