# Supplementary material for: Single extracellular vesicle surface protein‐based blood assay identifies potential biomarkers for detection and screening of five cancers
Source: Mol Oncol. 2024 Jan 9;18(3):743–61. doi: 10.1002/1878-0261.13586 (PMC10920081; doi:10.1002/1878-0261.13586)
Supplement: Supplementary file 1 — Fig. S1 Percentage of total EVs expressing at least one of the transmembrane or GPI‐anchored proteins in this study. Fig. S2. Batch effect adjustment by ComBat‐seq package. Fig. S3. Normalization of EV protein expression data by trimmed mean of M‐values algorithm. Fig. S4. Volcano plots of DEPs (P‐adj < 0.05). Fig. S5. Protein expression in tissues based on the Human Protein Atlas database. Fig. S6. EV subpopulation distribution among five cancers and control. Fig. S7. EV subpopulation distribution between each cancer and control. Fig. S8. Post hoc analysis of power calculation for the classification model performance in the validation dataset, using PASS v15. Table S1. Demographic and epidemiological characteristics of all cancer patients and controls in this study. Table S2. List of 207 proteins on PBA panel. Table S3. List of 198 proteins detected in this study and those referred to as EV markers in the MISEV2018 guidelines. Table S4. MISEV2018‐based checklist for EV characterization. Table S5. DEPs between pan‐cancer (n = 100) and control (n = 100). Table S6. DEPs between each cancer (n = 20) and control (n = 100). Table S7. Results of ROC analysis of DEPs (AUC > 0.800) for pan‐cancer. Table S8. Results of ROC analysis of DEPs (AUC > 0.800) for each cancer. Table S9. DEPCs between pan‐cancer (n = 100) and control (n = 100). Table S10. Results of ROC analysis of DEPCs (AUC > 0.800) for pan‐cancer. Table S11. DEPCs between each cancer (n = 20) and control (n = 100). Table S12. Results of ROC analysis of DEPCs (AUC > 0.800) for each cancer. Table S13. Binomial test for each EV subpopulation distribution between pan‐cancer (n = 100) and control (n = 100). Table S14. Cluster‐specific proteins in clusters 7, 9, and 11 between colorectal cancer and control samples (P‐adj < 0.05 and avg.log2FC > 0). Table S15. Adjusted P‐values of DEPs for each cancer (n = 20) against the other four cancers (n = 80). Table S16. Adjusted P‐values of DEPCs for each cancer (n = 20) against [file MOL2-18-743-s001.zip › Supplementary Fig. S1~S8.pdf]

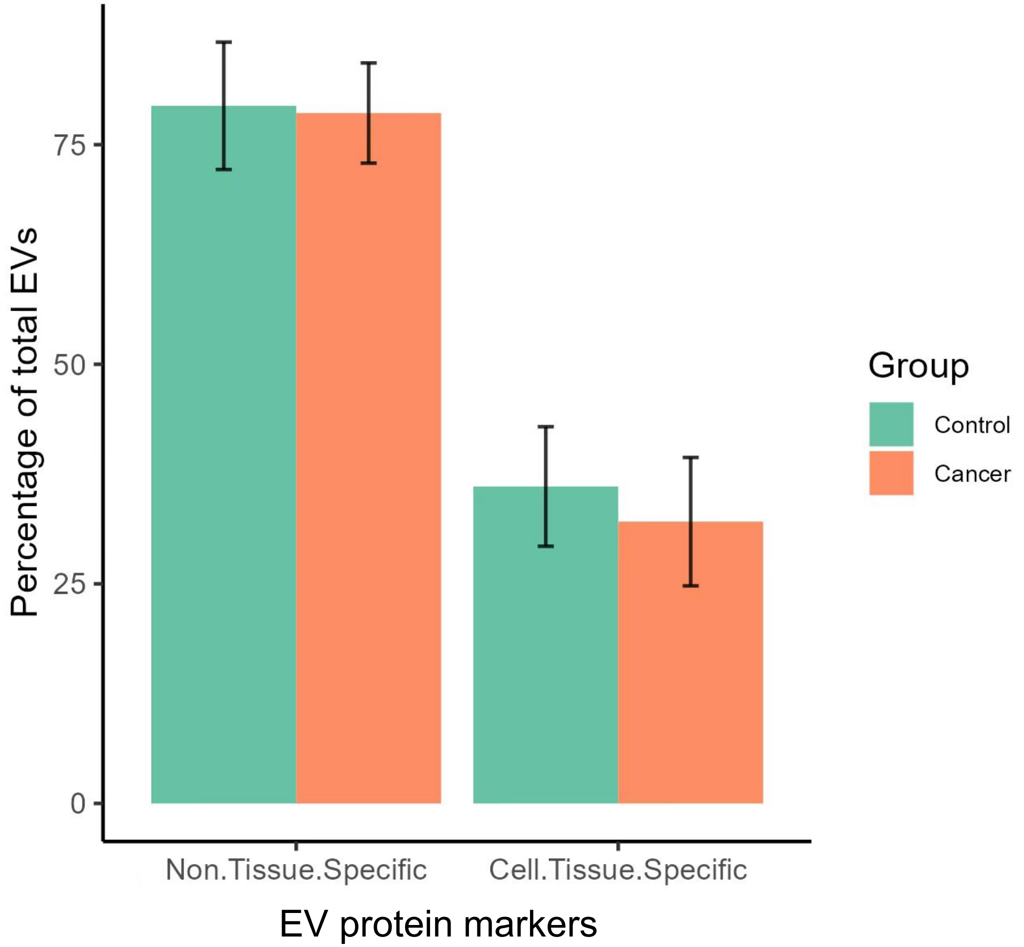

Supplementary Fig. S1

Percentage of total EVs expressing at least one of the transmembrane or GPI-anchored proteins in this study. This category of EV marker consists of non-tissue-specific and cell/tissue-specific proteins as outlined in the MISEV2018, including ADAM10, CD63, CD81, CD9, EPCAM, ERBB2, HLA-A, HLA-DRA, ITGA1, ITGA11, ITGA2, ITGA2B, ITGA3, ITGA4, ITGA4B7, ITGA5, ITGA6, ITGA8, ITGA9, ITGAL, ITGAM, ITGAV, ITGAX, ITGB1, ITGB2, ITGB3, ITGB4, ITGB5, ITGB6, ITGB7, ITGB8, LAMP1, LAMP2, NT5E, PECAM1, THY1. EVs: extracellular vesicles; GPI: glycosyl-phosphatidyl-inositol; MISEV2018: minimal information for studies of extracellular vesicles 2018.

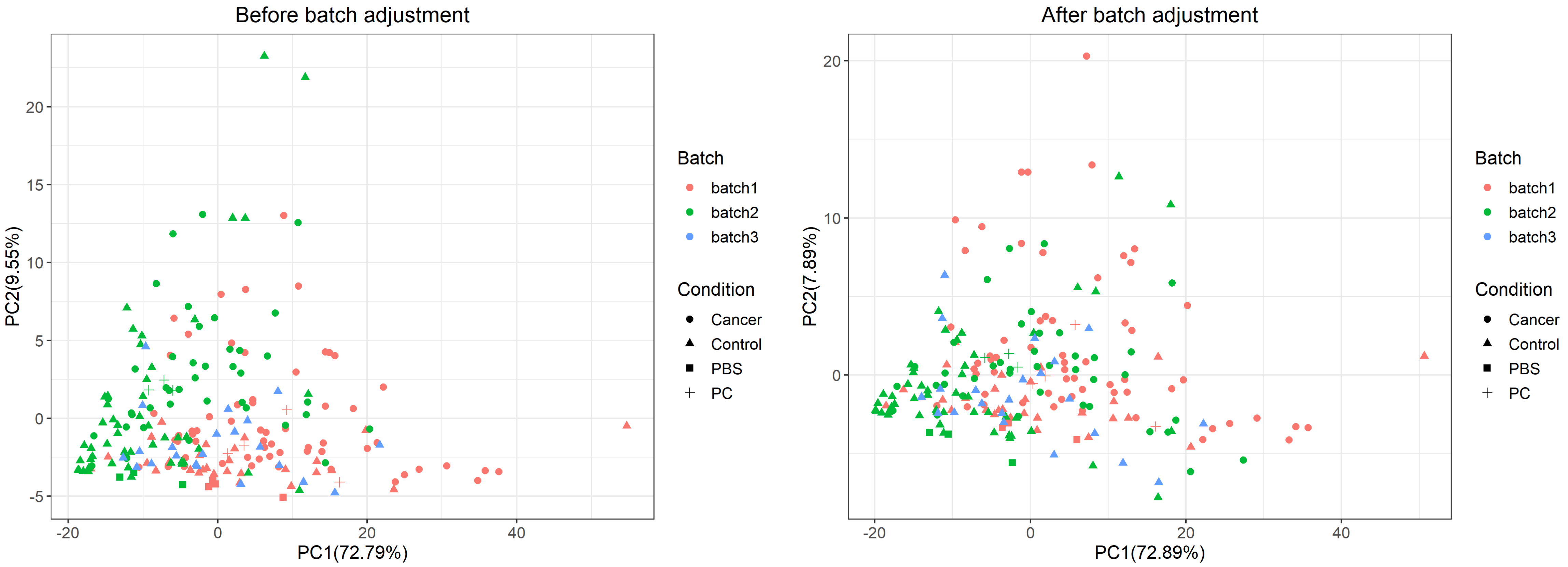

Supplementary Fig. S2

Batch effect adjustment by ComBat-seq package. PCA of plasma samples assayed on three batches before and after adjustment. PCA: principal component analysis; PBS: phosphate buffered saline; PC: positive control.

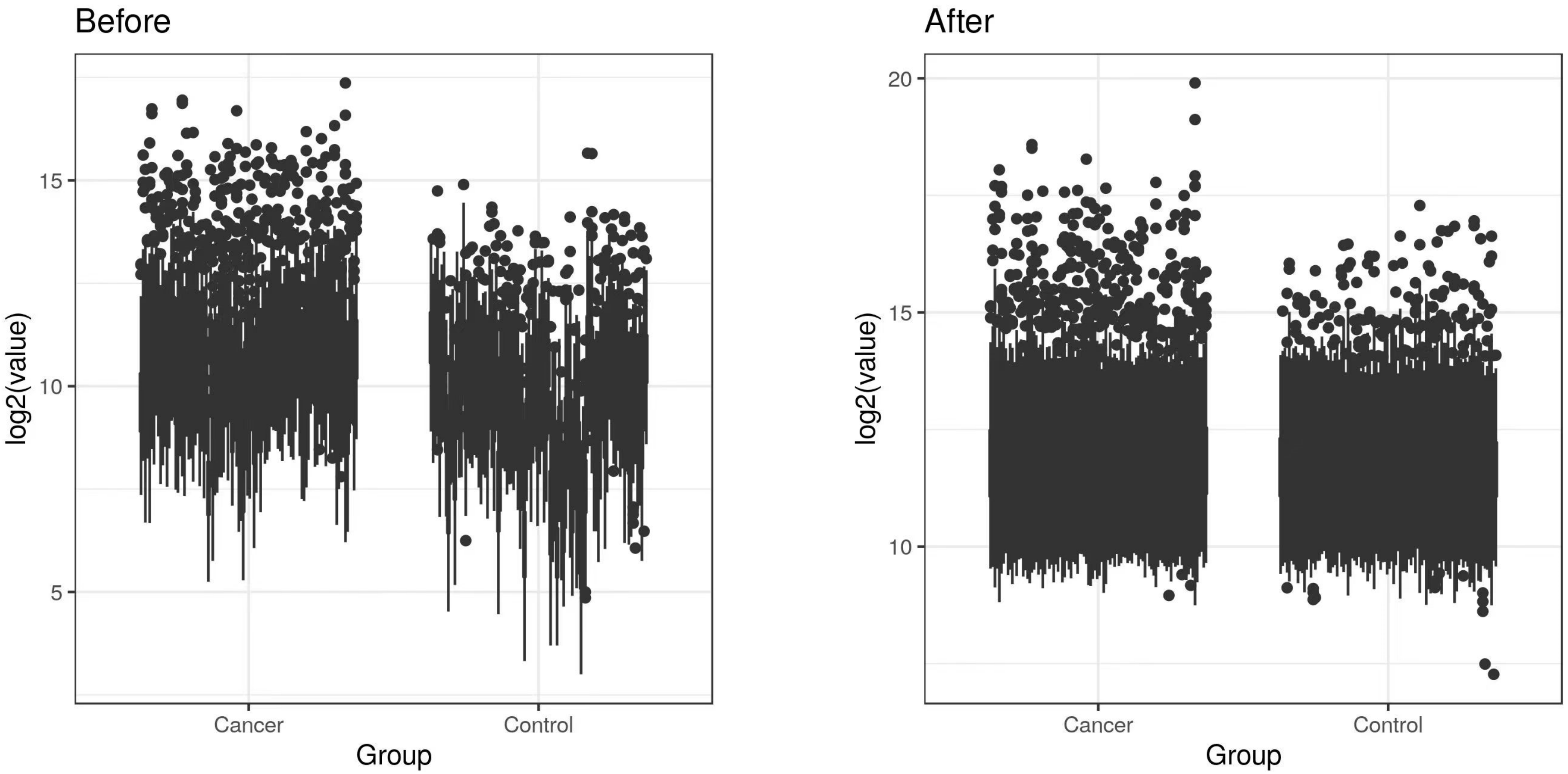

Supplementary Fig. S3
Normalization of EV protein expression data by trimmed mean of M-values algorithm. Comparison of EV protein expression level between cancer (n = 100) and control (n = 100) groups before and after normalization. EV: extracellular vesicle.

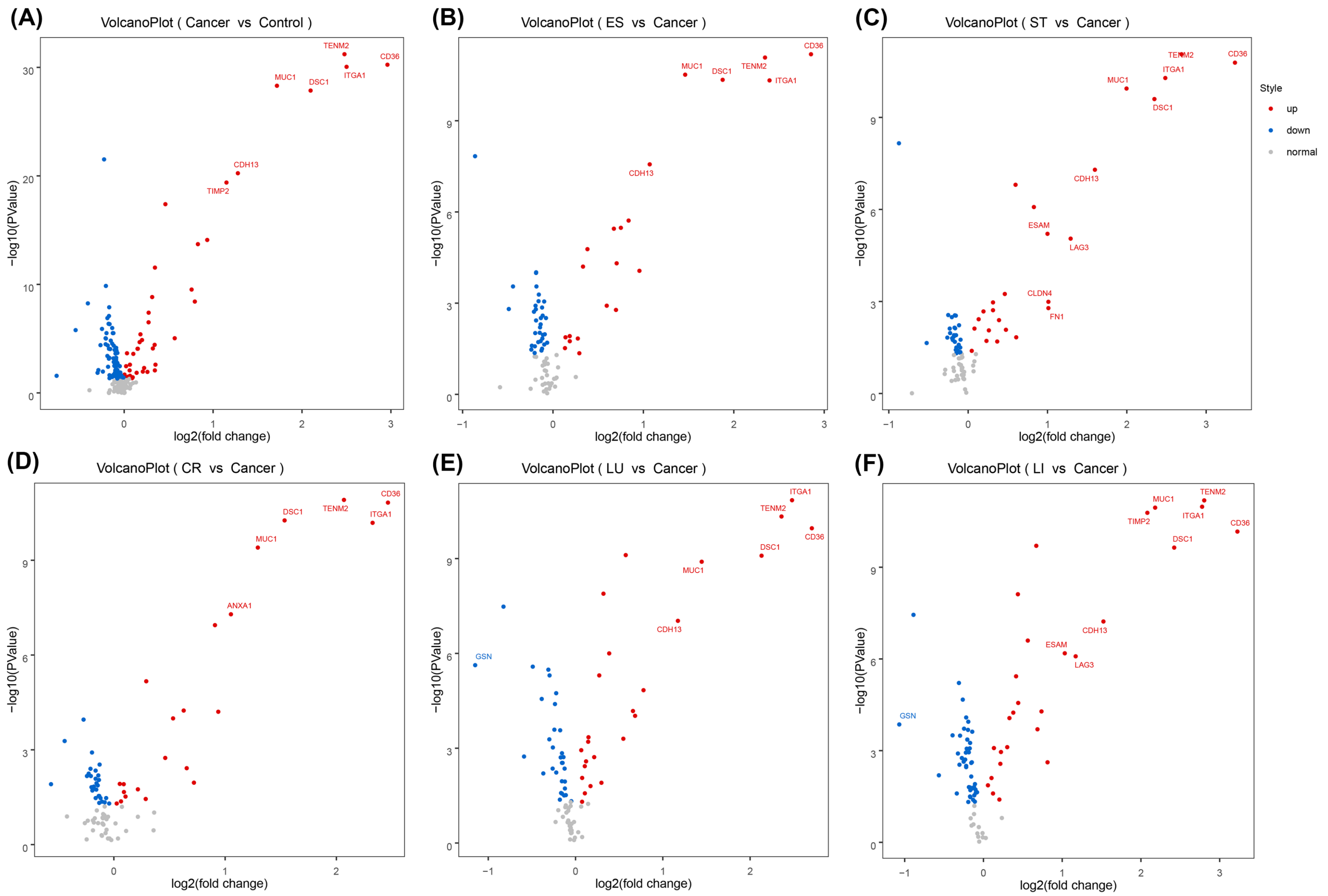

Supplementary Fig. S4

Volcano plots of DEPs (p-adj < 0.05) between (A) pan-cancer (n = 100) and control (n = 100) groups. (B) ES and control groups. (C) ST and control groups. (D) CR and control groups. (E) LI and control groups. (F) LU and control groups. B–F involves one cancer (n = 20) and control (n = 100) samples. DEPs: differentially expressed proteins; ES: esophageal cancer; ST: stomach cancer; CR: colorectal cancer; LI: liver cancer; LU: lung cancer

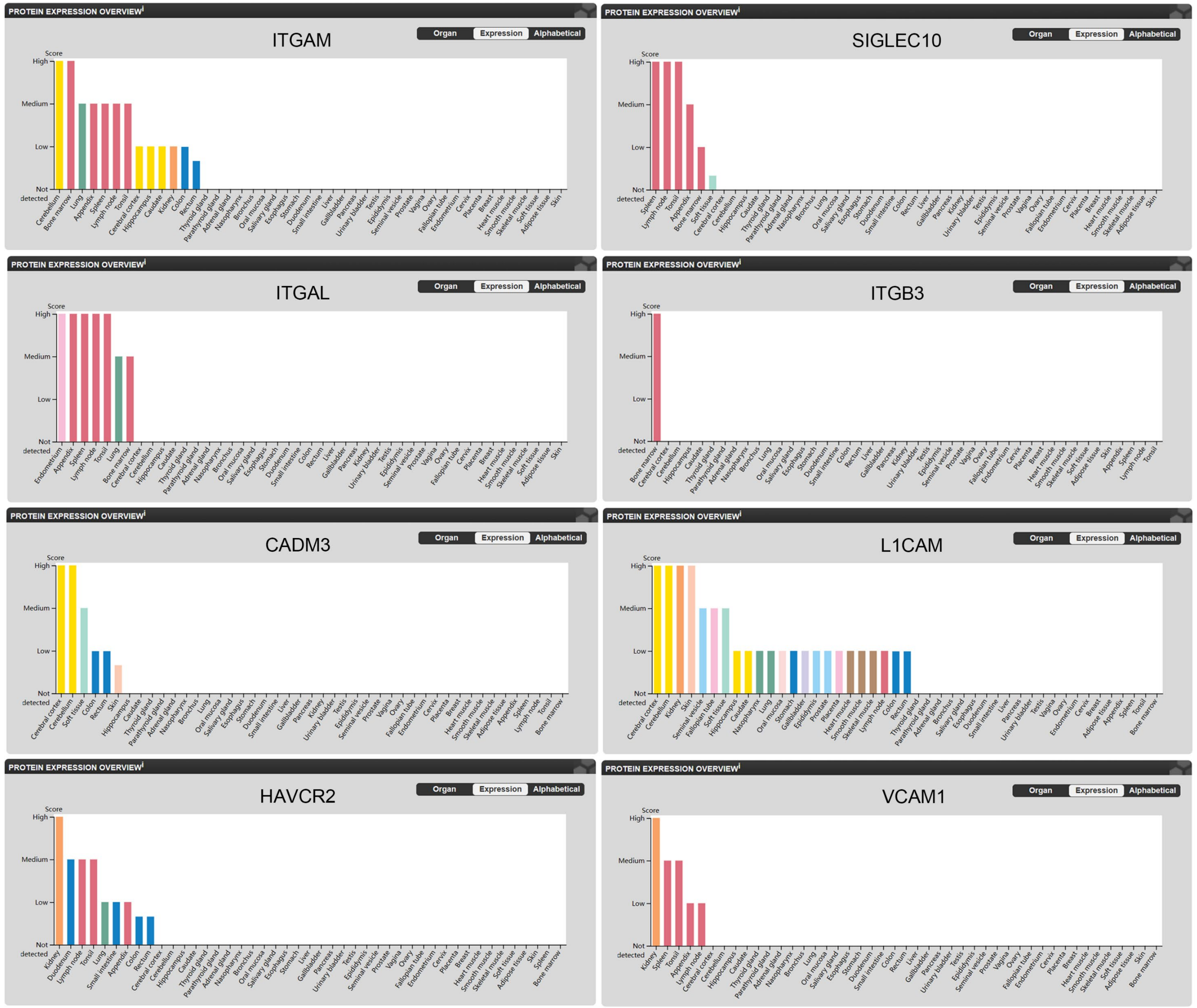

Supplementary Fig. S5

Protein expression in tissues based on the Human Protein Atlas database. Images available from v22.0.proteinatlas.org. The Human Protein Atlas database allow using images downloaded from the site when cited properly.

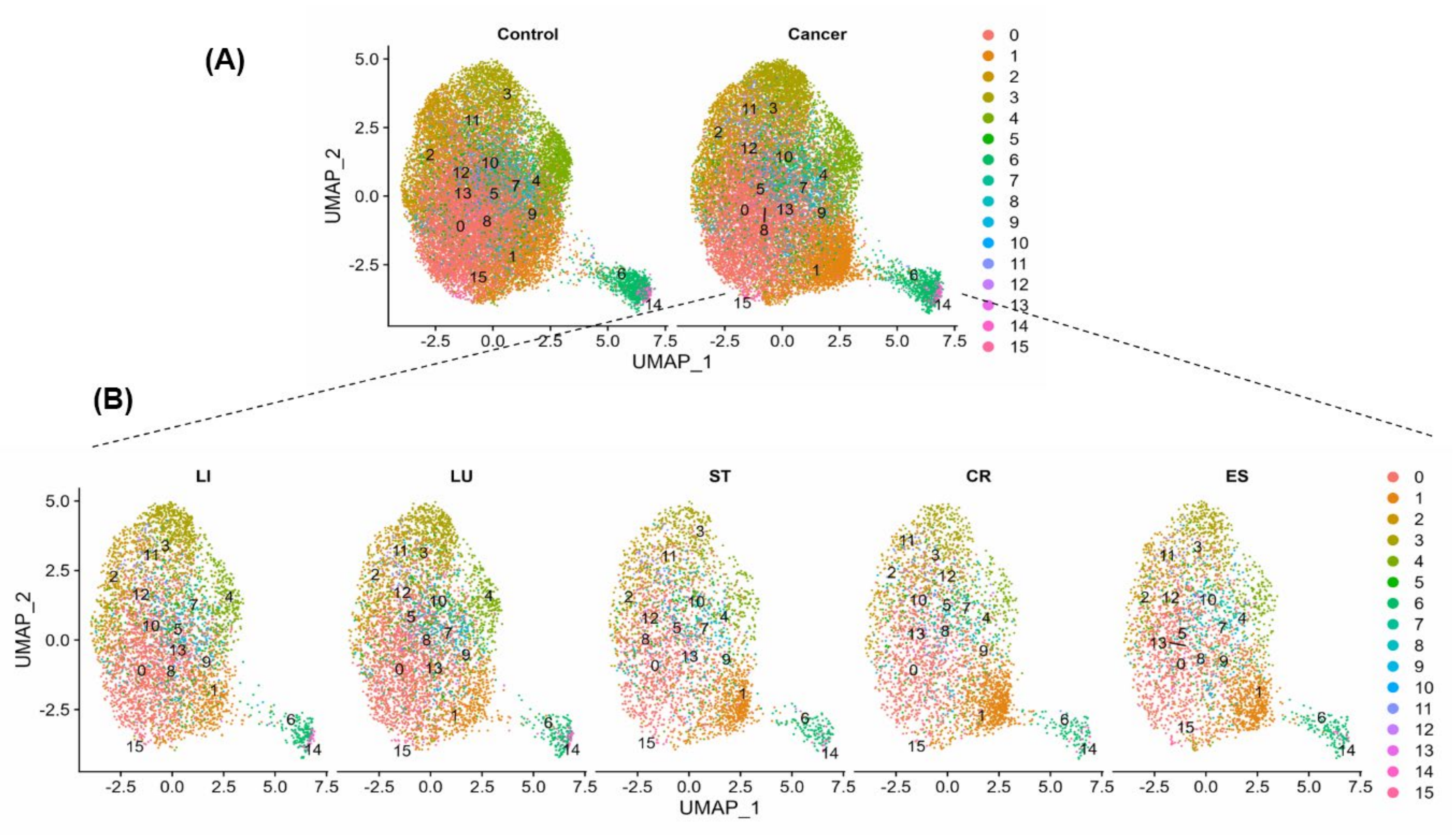

Supplementary Fig. S6

EV subpopulation distribution among five cancers and control. (A) A down-sampling strategy was applied to select 20,000 EVs from the cancer group (n = 100) and 20,000 EVs from the control group (n = 100), and (B) EVs from the cancer group were further classified by cancer type. EV: extracellular vesicle; ES: esophageal cancer; ST: stomach cancer; CR: colorectal cancer; LI: liver cancer; LU: lung cancer

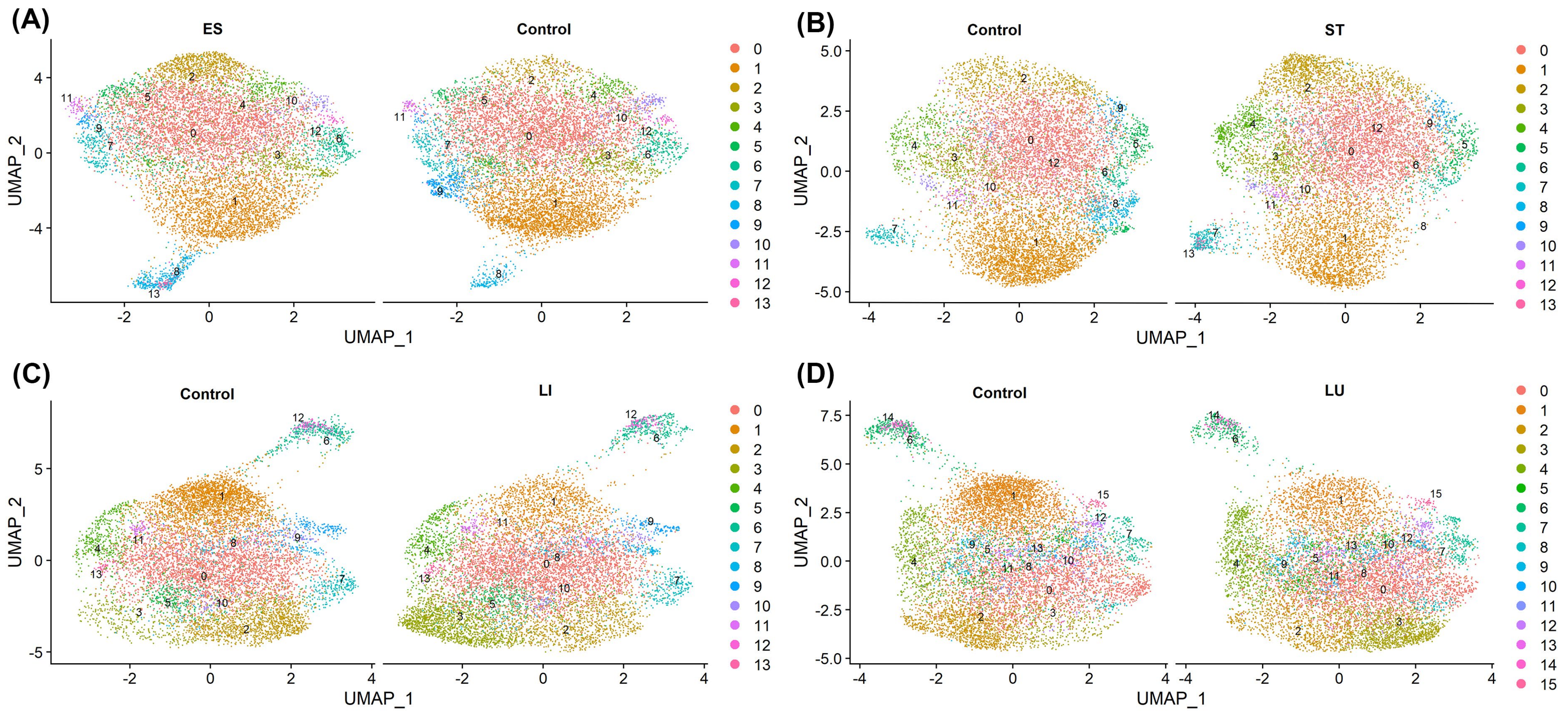

Supplementary Fig. S7

EV subpopulation distribution between each cancer and control. A down-sampling strategy was applied to select 10,000 EVs from each cancer group (*n* = 20) and 10,000 EVs from the control group (*n* = 100), respectively. (A) ES. (B) ST. (C) LI. (D) LU. EV: extracellular vesicle; ES: esophageal cancer; ST: stomach cancer; CR: colorectal cancer; LI: liver cancer; LU: lung cancer

Tests for One ROC Curve

Numeric Results for Testing AUC0 = AUC1 with Continuous Data
Test Type = Two-Sided. FPR1 = 0.00. FPR2 = 1.00. B = 1.00.

Power	N+	N-	N	AUC0'	AUC1'	Diff'	AUC0	AUC1	Diff	Alpha
0.79910	14	14	28	0.5000	0.7860	0.2860	0.5000	0.7860	0.2860	0.050

Supplementary Fig. S8
Post-hoc analysis of power calculation for the classification model performance in the validation dataset, using PASS v15. Power is the probability of rejecting a false null hypothesis; N+ and N- are the number of items sampled from each population; N is the total sample size, N+ + N-; AUC0' and AUC1' are the adjusted areas under the ROC curve for the null and alternative hypotheses, respectively; Diff' is AUC1 - AUC0. This is the adjusted difference to be detected; AUC0 and AUC1 are the actual areas under the ROC curve for the null and alternative hypotheses, respectively; Diff is AUC1 - AUC0; This is the difference to be detected; Alpha is the probability of rejecting a true null hypothesis; FPR1, FPR2 are the lower and upper bounds on the false positive rates; B is the ratio of the standard deviations of the negative and positive groups.
